# A meta-analysis indicating extra-short implants (≤ 6 mm) as an alternative to longer implants (≥ 8 mm) with bone augmentation

**DOI:** 10.1038/s41598-021-87507-1

**Published:** 2021-04-14

**Authors:** Xiaoran Yu, Ruogu Xu, Zhengchuan Zhang, Yang Yang, Feilong Deng

**Affiliations:** 1 Department of Oral Implantology, Hospital of Stomatology, Guanghua School of Stomatology, Sun Yat-Sen University, 56 Ling Yuan Xi Road, Guangzhou, 510006 Guangdong People’s Republic of China; 2 Guangdong Provincial Key Laboratory of Stomatology, 74 Zhong Shan Er Road, Guangzhou, 510006 Guangdong People’s Republic of China

**Keywords:** Dentistry, Dental implants

## Abstract

Extra-short implants, of which clinical outcomes remain controversial, are becoming a potential option rather than long implants with bone augmentation in atrophic partially or totally edentulous jaws. The aim of this study was to compare the clinical outcomes and complications between extra-short implants (≤ 6 mm) and longer implants (≥ 8 mm), with and without bone augmentation procedures. Electronic (via PubMed, Web of Science, EMBASE, Cochrane Library) and manual searches were performed for articles published prior to November 2020. Only randomized controlled trials (RCTs) comparing extra-short implants and longer implants in the same study reporting survival rate with an observation period at least 1 year were selected. Data extraction and methodological quality (AMSTAR-2) was assessed by 2 authors independently. A quantitative meta-analysis was performed to compare the survival rate, marginal bone loss (MBL), biological and prosthesis complication rate. Risk of bias was assessed with the Cochrane risk of bias tool 2 and the quality of evidence was determined with the Grading of Recommendations Assessment, Development, and Evaluation (GRADE) approach. 21 RCTs were included, among which two were prior registered and 14 adhered to the CONSORT statement. No significant difference was found in the survival rate between extra-short and longer implant at 1- and 3-years follow-up (RR: 1.002, CI 0.981 to 1.024, *P* = 0.856 at 1 year; RR: 0.996, CI 0.968 to 1.025, *P*  = 0.772 at 3 years, moderate quality), while longer implants had significantly higher survival rate than extra-short implants (RR: 0.970, CI 0.944 to 0.997, *P* < 0.05) at 5 years. Interestingly, no significant difference was observed when bone augmentations were performed at 5 years (RR: 0.977, CI 0.945 to 1.010, *P* = 0.171 for reconstructed bone; RR: 0.955, CI 0.912 to 0.999, *P* < 0.05 for native bone). Both the MBL (from implant placement) (WMD: − 0.22, CI − 0.277 to − 0.164, *P* < 0.01, low quality) and biological complications rate (RR: 0.321, CI 0.243 to 0.422, *P* < 0.01, moderate quality) preferred extra-short implants. However, there was no significant difference in terms of MBL (from prosthesis restoration) (WMD: 0.016, CI − 0.036 to 0.068, *P* = 0.555, moderate quality) or prosthesis complications rate (RR: 1.308, CI 0.893 to 1.915, *P* = 0.168, moderate quality). The placement of extra-short implants could be an acceptable alternative to longer implants in atrophic posterior arch. Further high-quality RCTs with a long follow-up period are required to corroborate the present outcomes.

*Registration number* The review protocol was registered with PROSPERO (CRD42020155342).

## Introduction

Dental implants have been widely applied to rehabilitation of edentulous jaws thanks to their acceptable clinical performance in clinical practice^1^. The success of dental implants relies on the adequate bone around the implants with favorable osseointegration. However, the vertical bone volume, one of the most essential limiting factors for dental implant placement and successful osseointegration, is insufficient frequently due to the inflammation, trauma or relatively rapid bone loss after tooth loss^2^.

Various surgical procedures have been employed for adequate vertical bone volume, such as bone grafts, sinus lifting, and nerve transposition. However, high technical sensitivity and considerable intra- and post-surgical complications contribute to the obstacles of these time-consuming and expensive surgical techniques. Thus, an alternative and less invasive therapy, the placement of short or extra-short implants, is popular with its easier procedure, less cost and quicker treatment^3–5^.

The initial definition for short implants is that the intra-bony lengths are less than 10 mm^6^, which has been fuzzy with the development of implant design and surface properties. The intra-bony lengths less than 10, 8, 7 and 6 mm all had been called as the short implants in various studies^7–10^. Besides, the concept named as ultrashort implants or extra-short implants which the intra-bony lengths are no more than 6 mm is gradually accepted^11,12^.

No consensus has been reached on the controversial issue that whether the length of implants is associated with their clinical outcomes^13–16^. The comparison between short implants (test group) and long implants (control group) may be improper when the influence of augmentation procedure, simultaneously or deferred with long implants placement, was not considered^14,17^. Implants in only one side of jaws (maxilla or mandible independently) or long implants placed in augmented bone exclusively were taken into consideration, which contributed to the limitations of some previous systematic reviews^18–26^. This article reported the outcomes for both jaws, and maxilla or mandible independently. Furthermore, outcomes were complemented by compromising the influence of the augmentation procedure.

In recent studies, implants with the length of 8 mm displayed comparable survival rates to longer implants, and a statistically significantly higher survival rate compared with the 6 mm implants^17,27^. This may suggest us that the implants of 8 mm length should be considered as predictable successful implants^17,28^. Implants with length more than 8 mm or 10 mm were frequently identified as control group in previous systematic reviews^2,29^. Nevertheless, dental implants with length of 8 mm or more (≥ 8 mm) in native or reconstructed bone are considered as control group compared with the extra-short implants (≤ 6 mm) for both and each jaw independently in present review, which has been seldomly performed before. In other words, we tend to focus on the clinical outcomes of extra-short (≤ 6 mm) implants by comparing with the longer (≥ 8 mm) implants, which were considered as the control group with acceptable clinical results. Only the RCTs taking extra-short (≤ 6 mm) implants as test group were included. While, the purpose of those RCTs, which compared the implants with length of 7 mm and longer implants, did not agree with the aim of this review. Therefore, the implants with length of 7 mm were not included since they were neither the research subjects nor the compared implants.

This systematic review aimed to compare the survival rate of extra-short implants (≤ 6 mm) and longer implants (≥ 8 mm) in edentulous jaws at different follow up. In addition, marginal bone loss, biological and prosthesis complication rate of extra-short vs longer implant was also evaluated. Furthermore, more essential details of included RCTs, such as the adherence to the CONSORT statement, prior registration and statistical issues were also concerned in this review.

## Materials and methods

This systematic review was conducted by following the Assessment of Multiple Systematic Reviews guidelines (AMSTAR 2)^30^ and the PRISMA (Preferred Reporting Items for Systematic Review and MetaAnalyses) Statement^31^. In addition, the review protocol was registered with the PROSPERO International Prospective Register of Systematic Reviews under the identification number CRD42020155342.

### Search strategy

Two reviewers conducted electronic systematic literature searches independently through PubMed, Web of Science, EMBASE and the Cochrane Library databases (until November 2020) using the following search terms: (a) Pubmed: ((short) OR (extra-short) OR (ultra-short)) AND ((implant) OR (implants) OR (dental implant) OR (dental implants)) AND (clinicaltrial[Filter]); (b) EMBASE: 'short implants':ti,ab,kw OR 'short dental implants':ti,ab,kw OR 'short implant':ti,ab,kw OR 'short dental implant':ti,ab,kw; (c) Web of Science: TOPIC: (short implant) OR TOPIC: (short implants)OR TOPIC: (short dental implants) ORTOPIC: (short dental implant) Refined by: Databases: (WOS) ANDDOCUMENT TYPES: (CLINICAL TRIAL); (d) the Cochrane Library databases: short implants in Title Abstract Keyword OR short dental implants in Title Abstract Keyword—in Trials (Word variations have been searched)—Source: CT.gov. Moreover, a thorough hand-searching incorporated the related journals and grey literature (from January 2016 to November 2020) supplemented by references within the retrieved articles.

### PICOS (patient, intervention, comparison, outcome, study design)

According to the PICOS format, a specific answerable question was illustrated as follows:

(P) Patients: Patients who received at least one extra-short dental implant (≤ 6 mm) or longer implant (≥ 8 mm) with or without bone augmentation followed for ≥ 12 months. Gender, nationality and race of patients are not restricted.
(I) Intervention: One or more extra-short (≤ 6 mm) implants placed in the maxilla and/or mandible.
(C) Comparison: One or more longer (≥ 8 mm) implants placed with or without bone augmentation in the maxilla and/or mandible.
(O) Outcome: Survival rate, marginal bone loss, biological and prosthesis complication rate between extra-short implants (≤ 6 mm) and longer length implants (≥ 8 mm) with or without bone augmentation in the maxilla and/or mandible.
(S) Study design: Randomized controlled trials.

### Eligibility criteria

Studies meeting the following predetermined inclusion criteria should be eligible: (a) Randomized controlled trials (RCTs) with an observation period of ≥ 12 months from implant placement; (b) human subjects receiving at least one extra-short (≤ 6 mm) implant(s) (test group) vs longer (≥ 8 mm) implant(s) (control group); (c) fixed prostheses was used as final restorations. (d) The survival rate of extra-short implant (≤ 6 mm) compared with longer implant (≥ 8 mm) were considered as the primary outcome which should be available in all the included studies. In addition, secondary outcomes comprised difference in marginal bone loss (MBL), supplemented with biological and prosthesis complication rates in this review.

### Study selection and data extraction

Initially, titles and abstracts of all studies were scanned and excluded by two reviewer authors independently and in duplicate. Full-text reading were required for further information to confirm the eligibility and fulfill the predetermined data extraction form in the final stage of screening. The data from each included study, such as number of implants, patient characteristics, implant characteristics, surgical procedure, was presented in Table 1. All disagreements were resolved by discussion or consulting a third author.

**Table 1:** Characteristics of included studies.

Number#	Study characteristics	Number of implants (patients)		Implants characteristics		Surgical procedures		Population characteristics	Prosthetic parameters
	1. Follow the CONSORT statement	1. Total		1. Length (mm)				1. Age (mean ± SD or mean, range)	1. Loading method
	2. Registration identifier	2. Maxillary		2. Diameter (mm)				2. Inclusion of heavy smokers	2. Retention method
Author (publication year)	3. Prior registered	3. Mandibular		3. Implant system				3. Smoker percentage (short/long)	3. Prothesis
Study design	4. Simple size calculation	Short	Long	Short	Long	Short	Long	4. History of periodontitist	
1# Guida et al. 2020	1. Yes	1. n = 75 (15)	1. n = 75 (15)	1. 6	1. 11	Implant placement	Implant placement	1. 63 ± 7.5	1. 3 months
3-year RCT	2. Clinical‐Trials. gov NCT03509402	2. n = 0	2. n = 0	2. 4	2. 4			2. Yes	2. Screwed
	3. No	3. n = 75 (15)	3. n = 75 (15)	3. OsseoSpeed TX, Astra Tech	3. OsseoSpeed TX, Astra Tech			3. 33%/40%	3. 5 implants-full arch
	4. Yes							4. NC	
2# Gulje et al. 2019	1. NC	1. n = 21 (20)	1. n = 20 (18)	1. 6	1. 11	Implant placement	Sinus floor elevation	1. NC	1. 3 months
5-year RCT	2. NC	2. n = 21(20)	2. n = 20 (18)	2. NC	2. NC			2. NC	2. Cemneted
	3. No	3. n = 0	3. n = 0	3. NC	3. NC			3. NC	3. Single crown
	4. NC							4. NC	
3# Weerapong et al. 2019	1. No	1. n = 23 (23)	1. n = 23 (23)	1. 6	1. 10	Implant placement	Implant placement	1. 51 (20–64)	1. Immediate
1-year RCT	2. No	2. n = 0	2. n = 0	2. NC	2. NC			2. Yes	2. Cemented
	3. No	3. n = 23 (23)	3. n = 23 (23)	3. PW + Dental Implant System	3. PW + Dental Implant System			3. NC	3. Single crown
	4. No							4. No	
4# Shi et al. 2019	1. No	1. n = 74 (75)	1. n = 143 (145)	1. 6	1. 8 or 10	Implant placement	Transcrestal sinus lift	1. 40.6	1. 3 months
1-year RCT	2. Clinical‐Trials.gov NCT02350075	2. n = 74 (75)	2. n = 143 (145)	2. 4.8 or 4.1	2. 4.8 or 4.1 or 3.3			2. No	2. Cemented
	3. Yes	3. n = 0	3. n = 0	3. Straumann Standard Plus	3. Straumann Standard Plus			3. NC	3. Singe or FPD
	4. Yes							4. Yes	
5# Bernardi et al. 2018	1. No	1. n = 86 (36)	1. n = 84 (36)	1. 6	1. 10	Implant placement	Vertical bone augmentation	1. 62(43–77)	1. 2 months
1-year RCT	2. No	2. n = 0	2. n = 0	2. 4.1	2. 3.9			2. NC	2. Screwed
	3. No	3. n = 86 (36)	3. n = 84 (36)	3. IM Macon, MACODENTALCARE	3. ConicalActive, MACODENTALCARE			3. NC	3. Single crown
	4. No							4. NC	
6# Bolle et al. 2018	1. Yes	1. n = 80 (40)	1. n = 87 (40)	1. 4	1. 10 or 11.5 or 13	Implant placement	Maxilla: lateral sinus floor elevation	1. 61.3(46–73)	1. 4 months
1-year RCT	2. No	2. n = 37 (20)	2. n = 43 (20)	2. 4 or 4.5	2. 4		Mandible: vertical bone augmentation	2. Yes	2. Screwed or cemented
	3. No	3. n = 41 (20)	3. n = 46 (20)	3. TwinKon Universal SA2	3. TwinKon Universal SA2			3. 10%/40%	3. Single crown or FPF
	4. No							4. Yes	
7A# Felice et al. 2018	1. Yes	1. n = 80 (40)	1. n = 91 (40)	1. 6	1. 11.5 or 13 or 15	Implant placement	Maxilla: lateral sinus floor elevation	1. 55.9 (42–80)	1. 4 months
7B# Felice, Pistilli, et al. 2019	2. No	2. n = 39 (20)	2. n = 44 (20)	2. 4	2. 4		Mandible: vertical bone augmentation	2. Yes	2. Screwed or cemented
5-year RCT	3. No	3. n = 41 (20)	3. n = 47 (20)	3. Southern implants	3. Southern implants			3. 12.5%/10%	3. FPF
	4. Yes							4. Yes	
8A# Gastaldi et al. 2018	1. Yes	1. n = 68 (40)	1. n = 68 (40)	1. 5	1. 11.5 or 13 or 15	Implant placement	Maxilla: lateral sinus floor elevation	1. 55.3 (39–80)	1. 4 months
8B# Esposito et al. 2019	2. No	2. n = 36 (20)	2. n = 37 (20)	2. 5	2. 5		Mandible: vertical bone augmentation	2. Yes	2. Screwed or cemented
5-year RCT	3. No	3. n = 32 (20)	3. n = 31 (20)	3. ExFeel, MegaGen	3. ExFeel, MegaGen			3. 15%/17.5%	3. Single crown or FPF
	4. No							4. Yes	
9# Rokn et al. 2018	1. Yes	1. n = 25 (11)	1. n = 22 (11)	1. 4	1. 8 or 10	Implant placement	Vertical bone augmentation	1. 50.3	1. 2 months
1-year RCT	2. No	2. n = 0	2. n = 0	2. 4.1	2. 4.1			2. NC	2. Screwed
	3. No	3. n = 25 (11)	3. n = 22 (11)	3. Straumann Standard Plus	3. Straumann Standard Plus			3. NC	3. Single crown or FPF
	4. Yes							4. Yes	
10# Shah et al. 2018	1. No	1. n = 25 (25)	1. n = 25 (25)	1. 6	1. 10	Implant placement	Vertical bone augmentation	1. 58.4 ± 11.6	1. 3 or 6 months
1-year RCT	2. No	2. NC	2. NC	2. NC	2. NC			2. Yes	2. NC
	3. No	3. NC	3. NC	3. MIS seven	3. MIS seven			3. 8%/12%	3. NC
	4. Yes							4. Yes	
11A# Gulje et al. 2020	1. Yes	1. n = 108 (49)	1. n = 101 (46)	1. 6	1. 11	Implant placement	Implant placement	1. 54.5(26–70)	1. 6 weeks
11B# Zadeh et al. 2018	2. Clinical‐Trials.gov NCT00545818	2. NC	2. NC	2. 4	2. 4			2. No	2. Screwed
11C# Gulje et al. 2013	3. Yes	3. NC	3. NC	3. OsseoSpeed	3. OsseoSpeed			3. 40.8%/28.3%	3. FPD (by 2–3 implants)
5-year RCT	4. Yes							4. Yes	
12A# Sahrmann et al. 2016	1. Yes	1. n = 40 (40)	1. n = 46 (46)	1. 6	1. 10	Implant placement	Transcrestal sinus lift	1. 58.2 ± 12.8	1. 10 weeks
12B# Naenni et al. 2018	2. German Clinical Trials DRKS00006290	2. n = 12 (12)	2. n = 22 (22)	2. 4.1	2. 4.1			2. Yes	2. Screwed
5-year RCT	3. No	3. n = 28 (28)	3. n = 24 (24)	3. Straumann Standard Plus	3. Straumann Standard Plus			3. 55%/47.8%	3. Single crown
	4. Yes							4. Yes	
13A# Schincaglia et al. 2015	1. No	1. n = 67 (50)	1. n = 70 (51)	1. 6	1. 11 or 13 or 15	Implant placement	Lateral sinus floor elevation	1. 55.7(20–77)	1. 6–7 months
13B# Pohl et al. 2017	2. Clinical‐Trials.gov NCT01030523	2. n = 67 (50)	2. n = 70 (51)	2. 4	2. 4			2. NC	2. Screwed or cemented
13C# Thoma et al. 2018	3. No	3. n = 0	3. n = 0	3. OsseoSpeedTM 4.0S, Astra Tech	3. OsseoSpeedTM 4.0S, Astra Tech			3. 26%/55%	3. Single crown
5-year RCT	4. Yes							4. Yes	
14# Bechara et al. 2017	1. Yes	1. n = 45 (33)	1. n = 45 (20)	1. 6	1. 10 or 11.5 or 13 or 15	Implant placement	Lateral sinus floor elevation	1. 48.1 ± 15.1	1. 4 months
3-year RCT	2. No	2. n = 45 (33)	2. n = 45 (20)	2. 4–8	2. 4–8			2. NC	2. Screwed or cemented
	3. No	3. n = 0	3. n = 0	3. AnyRidge Implants, MegaGen	3. AnyRidge Implants, MegaGen			3. 21.2%/40%	3. Single crown or FPF
	4. No							4. Yes	
15A# Felice et al. 2015	1. Yes	1. n = 16 (10)	1. n = 18 (10)	1. 5 or 6	1. 10	Implant placement	Lateral sinus floor elevation	1. 56 (43–70)	1. 4 months
15B# Gastaldi et al. 2017	2. No	2. n = 16 (10)	2. n = 18 (10)	2. 5	2. 6			2. Yes	2. Screwed or cemented
3-year RCT	3. No	3. n = 0	3. n = 0	3. NXFOS5/6xx, Zimmer Biomet	3. NXFOS5/6xx, Zimmer Biomet			3. 40%/70%	3. Single or FPD
	4. No							4. Yes	
16# Cannizzaro et al. 2015	1. Yes	1. n = 152 (30)	1. n = 151 (30)	1. 5	1. 11.5	Implant placement	Implant placement	1. 55.9 (48–80)	1. Immediate
1-year RCT	2. No	2. n = 90 (15)	2. n = 91 (15)	2. 5	2. 5			2. Yes	2. Screwed
	3. No	3. n = 62 (15)	3. n = 60 (15)	3. Supershort NanoTite	3. Supershort NanoTite			3. 40%/33%	3. Crossarch prosthesis
	4. No							4. Yes	
17# Gulje et al. 2014	1. Yes	1. n = 21 (21)	1. n = 20 (20)	1. 6	1. 11	Implant placement	Lateral sinus floor elevation	1. 49 (29–72)	1. 3 months
1-year RCT	2. No	2. n = 21 (21)	2. n = 20 (20)	2. 4	2. 4			2. No	2. Cemented
	3. No	3. n = 0	3. n = 0	3. OsseoSpeed 4.0 S, Astra Tech	3. OsseoSpeed 4.0 S, Astra Tech			3. No	3. Single crown
	4. Yes							4. Yes	
18# Romeo et al. 2014	1. Yes	1. n = 26 (11)	1. n = 28 (13)	1. 6	1. 10	Implant placement	Implant placement	1. 53 (32–75)	1. 6 weeks
5-year RCT	2. No	2. n = 5	2. n = 7	2. 4.1	2. 4.1			2. No	2. Screwed
	3. No	3. n = 21	3. n = 21	3. Straumann, Basel	3. Straumann, Basel			3. 27.3%/38.5%	3. NC
	4. Yes							4. Yes	
19A# Esposito et al. 2011	1. Yes	1. n = 60 (30)	1. n = 68 (30)	1. 5	1. 10	Implant placement	Maxilla: lateral sinus floor elevation	1. 56 (37–70)	1. 4 months
19B# Esposito et al. 2014	2. No	2. n = 34 (15)	2. n = 38 (15)	2. 6	2. 6		Mandible: vertical bone augmentation	2. Yes	2. Screwed
19C# Felice, Barausse et al. 2019	3. No	3. n = 26 (15)	3. n = 30 (15)	3. Rescue /EZ Plus MegaGen	3. Rescue /EZ Plus MegaGen			3. 20%/20%	3. NC
5-year RCT	4. Yes							4. Yes	
20# Pistilli et al. 2013	1. Yes	1. n = 68 (40)	1. n = 68 (40)	1. 5	1. 10 or 11.5 or 13 or 15	Implant placement	Maxilla: Lateral sinus floor elevation	1. 55.3 (39–80)	1. 4 months
1-year RCT	2. No	2. n = 36 (20)	2. n = 32 (20)	2. 5	2. 5		Mandible: Vertical bone augmentation	2. Yes	2. Screwes or cemented
	3. No	3. n = 32 (20)	3. n = 31 (20)	3. ExFeel, MegaGen	3. ExFeel, MegaGen			3. 35%/30%	3. NC
	4. No							4. Yes	
21# Rossi et al. 2016	1. No	1. n = 30 (30)	1. n = 30 (30)	1. 6	1. 10	Implant placement	Implant placement	1. 48.1	1. 6 weeks
5-year RCT	2. No	2. n = 12 (12)	2. n = 15 (15)	2. 4.1	2. 4.1			2. NC	2. NC
	3. No	3. n = 18 (18)	3. n = 15 (15)	3. Straumann AG	3. Straumann AG			3. 20%/23.3%	3. Single crown
	4. No							4. NC	

NC, not clear, not reported; FPD, fixed partial denture; RCT, Randomized controlled trail; CONSORT, Consolidated Standards of Reporting Trials.

### Statistical analysis

Only the studies made similar comparisons reporting the same outcomes, could a meta-analysis be conducted by software Stata version 15 (StataCorp. 2017. Stata Statistical Software: Release 15. College Station, TX: StataCorp LLC). The main effect size measure for quantitative continuous data (MBLs) was considered weighted mean difference (WMD). The quantitative binary data (implant survival rate, biological and prosthesis complication rate) were evaluated using risk ratio (RR). Inverse Variance methods and Mantel–Haenszel were used as the Weighting Methods for WMD and RR, respectively. By definition, RR < 1 indicated a lower event rate of test group, and WMD < 0 indicated a lower MBL was observed in test group.

Q Cochrane test, the related *P* values, *I*^2^ and the 95% confidence intervals for *I*^2^ were used to evaluate the heterogeneity. Summary estimates of RR were calculated by random-effects models if heterogeneity was proved to be high (*P* < 0.05, *I*^2^ > 50%)^32^. According to the recommendations of Higgins^33^, subgroup analysis, meta-regression, sensitivity testing and exploration of publication bias were conducted to investigate the heterogeneity, and the significance was set at *P* < 0.05. Subgroup analyses were performed to test the effect of bone augmentation procedure. Meta-regression analyses were performed to test categorical variables such as loading method (immediate/early/conventional), and exclusion or inclusion of heavy smokers. The effect of smoking habits on the clinical outcomes was investigated through the smoking percentage ratio between short and long implant groups (S/L) and the total percentage of smokers. Additionally, one-out-removed method was performed for the sensitivity analysis. Funnel plots and Egger tests were implemented to assess the probability of publication bias.

### Risk of bias and quality of evidence

The methodological quality assessment of included articles was undertaken by two investigators based on the published full-text articles independently using the Cochrane risk of bias 2 (ROB 2) assessment tool for RCTs^34^. In case of disagreement, it was solved by a discussion with the third author. ROB 2, focusing on different aspects of trial design, conduct and reporting, is structured into a fixed set of domains of bias, which include a series of questions (‘signalling questions’) for elicit information about features of the trial that are relevant to risk of bias. A judgement arising from each domain, which could be ‘Low’, ‘High’ risk of bias, or ‘Some concerns’ about the risk of bias, is proposed based on answers to the signalling questions.

The Grades of Recommendation, Assessment, Development and Evaluation (GRADE) tool has been used to summarise the overall quality of the evidence^35^. Issues with bias, inconsistency, imprecision, indirectness and publication bias can decrease certainty, whereas large effect, plausible confounding and dose response can increase.

## Results

### Study selection

**Figure 1:**
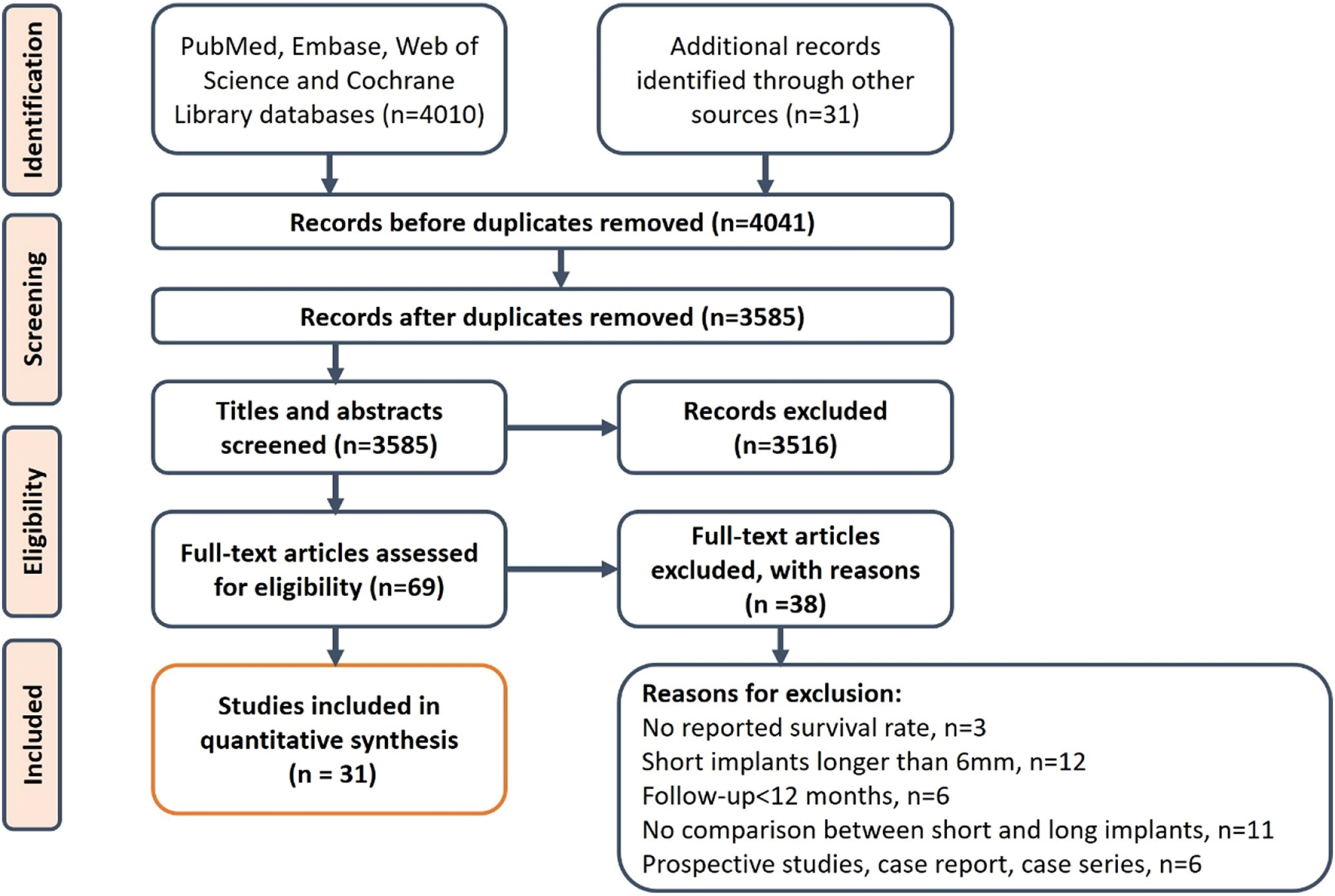
PRISMA flowchart of the screening process.

Electronic searches identified a total of 4010 publications including 1757 from PubMed, 1387 from Web of Science, 570 from EMBASE, 296 from the Cochrane Central Register of Controlled Trials. Furthermore, an additional 31 articles were collected through manual screening. After removal of duplicates and screening the title and abstract, 69 publications were selected. After full-text screening, 38 articles were excluded for reasons (Fig. 1, Supplementary file 2), leaving a total of 31 articles with different follow up times for inclusion in the following statistical analysis and interpretation. While, articles which reported the outcomes of the same RCT at different follow-up would be counted as the same study. Therefore, the 31 included articles were categorized into 21 series of studies and each series reported the outcomes of one independent RCT.

### Risk of bias and quality of evidence

The methodological quality assessment of 21 series of studies was undertaken by ROB 2 tool and shown in Table 2 and Supplementary Figure 3. Five studies were considered as having a high risk of bias, and judgments expressed “some concern” in seven studies, while the remaining were characterized by a low risk of bias. According to the GRADE system, pooling of studies on implants survival rate, MBL and complications rate provided low- to moderate-quality evidence in Table 3.

**Table 2:**
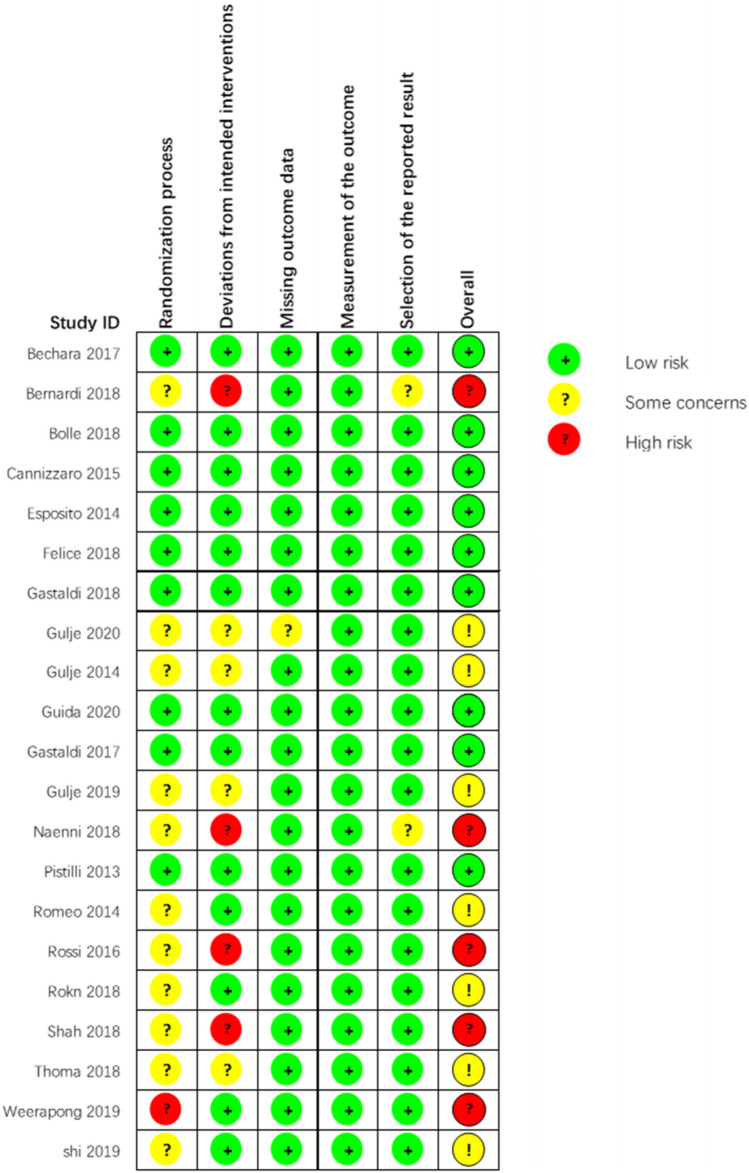
Quality assessment of included studies by ROB 2.

Generated by RevMan Web, https://revman.cochrane.org.

**Table 3:** Grades of recommendation, assessment, development and evaluation approach summarizing the evidence.

Certainty assessment							No of patients		Effect		Certainty	Importance
No. of studies	Study design	Risk of bias	Inconsistency	Indirectness	Imprecision	Other considerations	Extra-short implants (≤ 6 mm)	Longer implants (≥ 8 mm)	Relative (95% CI)	Absolute (95% CI)		
**Survival rate (implant level) (follow up: range 1 years to 5 years)**												
22	Randomised trials	Not serious	Not serious	Not serious	Serious^a^	None	1193/1243 (96.0%)	1290/1333 (96.8%)	RR 0.991 (0.974 to 1.009)	9 fewer per 1000 (from 25 fewer to 9 more)	⨁⨁⨁◯ Moderate	Critical
**MBL (measured from IP) (follow up: range 1 years to 5 years)**												
19	Randomised trials	Not serious	Serious^b^	Not serious	Serious	None	962	953	–	MD 0.22 mm lower (0.277 lower to 0.164 lower)	⨁⨁◯◯ Low	Critical
**MBL (measured from PR) (follow up: range 1 years to 5 years)**												
13	Randomised trials	Not serious	Not serious	Not serious	Serious	None	404	485	–	MD 0.016 mm higher (0.036 lower to 0.068 higher)	⨁⨁⨁◯ Moderate	critical
**Biological complications rate (follow up: range 1 years to 5 years)**												
14	Randomised trials	Not serious	Serious^c^	Not serious	Not serious	None	50/460 (10.9%)	161/520 (31.0%)	RR 0.321 (0.243 to 0.422)	210 fewer per 1000 (from 234 to 179 fewer)	⨁⨁⨁◯ Moderate	Important
**Prosthesis complications rate (follow up: range 1 years to 5 years)**												
12	Randomised trials	Not serious	Not serious	Not serious	Serious^d^	None	53/386 (13.7%)	48/379 (12.7%)	RR 1.092 (0.777 to 1.535)	12 more per 1000 (from 28 fewer to 68 more)	⨁⨁⨁◯ Moderate	Important
**Survival rate (patient level) (follow up: range 1 years to 5 years)**												
18	Randomised trials	Not serious	Not serious	Not serious	Serious^d^	None	514/566 (90.8%)	592/632 (93.7%)	RR 0.970 (0.938 to 1.003)	28 fewer per 1000 (from 58 fewer to 3 more)	⨁⨁⨁◯ Moderate	Critical

Question: Extra-short implants (≤ 6 mm) compared to longer implants (≥ 8 mm) for partially or totally edentulous patients.

GRADE Working Group grades of evidence. High certainty: We are very confident that the true effect lies close to that of the estimate of the effect. Moderate certainty: We are moderately confident in the effect estimate: The true effect is likely to be close to the estimate of the effect, but there is a possibility that it is substantially different. Low certainty: Our confidence in the effect estimate is limited: The true effect may be substantially different from the estimate of the effect. Very low certainty: We have very little confidence in the effect estimate: The true effect is likely to be substantially different from the estimate of effect. Generated by GRADEpro GDT web application, http://gradepro.org.

CI: confidence interval; RR: risk ratio; MD: mean difference.

^a^Small simple size (less than OIS), CI of RR included 1.

^b^Heterogeneity across the studies, I^2^ = 58.10%, CI for I^2^ = 30.3% to 74.8%.

^c^Heterogeneity across the studies, I^2^ = 48.8%, CI for I^2^ = 5.1% to 72.39%.

^d^Small simple size (less than OIS), CI of RR included 1.

### Characteristics of the studies

The characteristics of the 31 included articles, which were categorized into 21 series of studies, are listed in Table 1. Overall, 2576 implants have been placed compromising 1243 extra-short implants and 1333 longer implants in 1387 patients. Subgroups were performed when maxillary and mandibular implants were reported as separate analysis units in the RCTs^5,36–56^, while 4 studies combined the outcomes of implants installed in both maxilla and mandible^12,57–59^. 7 out of 21 RCTs restored the implants with single-crown prostheses^36,38,40,41,50–52,54,56,60^, while 5 with splinted prostheses exclusively^43,45,47,57,59,61^ and the others with either a single-crown or splinted prostheses. Eight studies used only screw-retained prostheses^5,39,41,45,47,49,54,57–60,62^, while four studies applied only cement-retained prostheses^51–53,56^ and the others exerted both prosthetic retention techniques. When it comes to the loading protocol, an immediate loading method was utilized in two studies^47,52^, an early loading protocol (< 3 months) in four studies^5,41,50,54,57–60^ and the remaining conducted conventional loading methods (≥ 3 months)^12,36–40,42–46,48,49,51,53,55,56,61–64^. In addition, heavy smokers were recruited in 11 investigations (≥ 10 cigarettes/day)^12,37,39,41–49,52,59–63^, whereas excluded in the remaining studies.

19 selected articles employed the patient as the unit of analysis, while the outcomes of implant-level were reported in the remaining studies. Different approaches to address the within-patient correlation, which is very common in oral research since multiple sites within a single patient may be inappropriately considered as independent analysis units, were adopted in few studies^53,65^. Only one implant was installed in each patient in some studies resulting in the avoidance of the problem of within-patient correlation^12,41,52,56^. Nevertheless, survival rate without adjustment for within-patient correlation were reported in a few studies, which utilized all implants while ignored the dependence among implants from the same subject^59,66,67^.

### Publication bias

Funnel plots displayed the publication bias calculated by Egger test (Supplementary Figure 2). In MBL from prosthesis restoration (PR) and prosthetic complication rate, there was no evidence of publication bias, depending on the symmetrical funnel plot and Egger’s test (*P* = 0.894, *P* = 0.540, respectively). In the funnel plot of survival rate, MBLs from implant placement (IP) and biological complications, despite the pattern of heterogeneous points in funnel plots, the publication bias was negligible due to the result of Egger's test (*P* = 0.778, *P* = 0.223, *P* = 0.539, respectively).

### Implant survival rate

There were 31 studies included with different follow-up years, which revealed that the individual survival rate for the reported extra-short and longer implants throughout the studies was 95.98% and 96.77%, and the overall survival rate of the implants was 96.39%.

**Figure 2:**
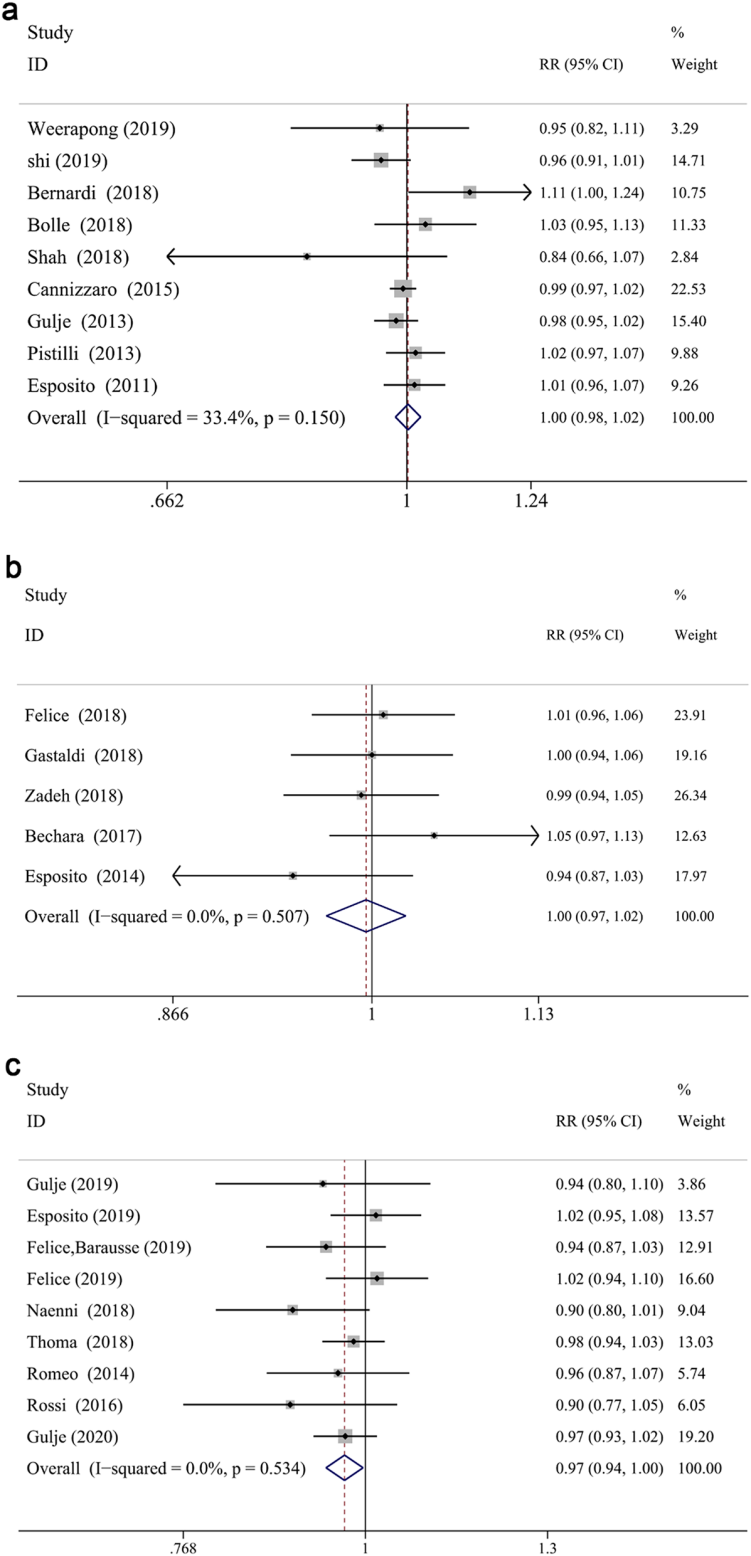
Forest plots (RR) of the survival rate comparing extra-short with longer implants group in 1-year (**a**), 3-years (**b**) and 5-years (**c**) results. Mantel–Haenszel (MH)- weighted RR < 1 indicated a lower survival rate of extra-short implants than the longer implants.

**Figure 3:**
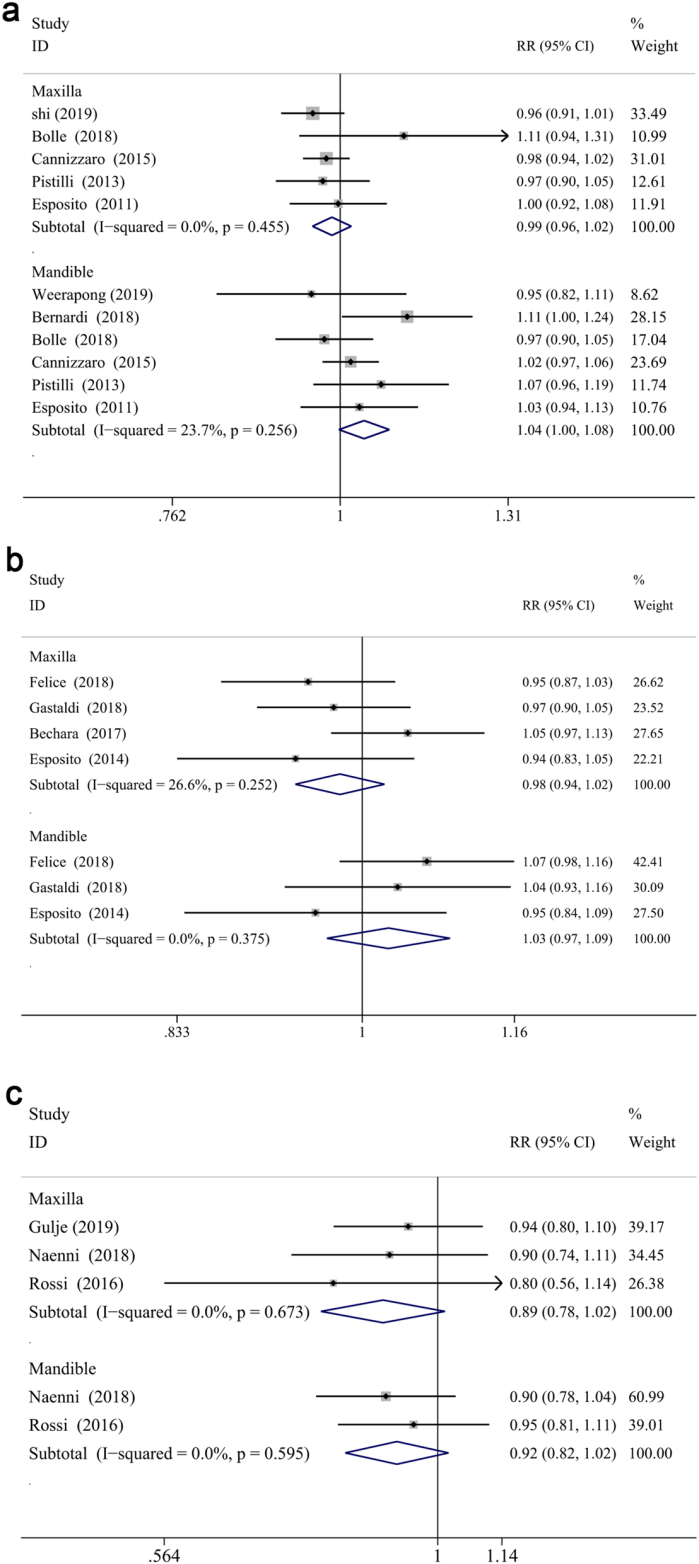
Subgroup analyses of maxilla/mandible on survival rate at 1-year (**a**), 3-years (**b**), 5-years (**c**) follow-up. Mantel–Haenszel (MH)-weighted RR < 1 indicated a lower survival rate of extra-short implants than the longer implants.

The meta-analysis revealed that the survival rate of extra-short and longer implants failed to prove a significantly statistical difference in both jaws at 1- and 3-year follow up (RR: 1.002, CI 0.981 to 1.024, *P* = 0.856 at 1 year; RR: 0.996, CI 0.968 to 1.025, *P* = 0.772 at 3 years, Fig. 2a,b). However, statistical significance was demonstrated in the survival difference between two groups at 5-years follow up (RR: 0.970, CI 0.944 to 0.997, *P* < 0.05) (Fig. 2c), which proved that longer implants have a higher survival rate than extra-short implants in longer follow up periods. While, no significant difference was found between two groups in the maxilla (RR: 0.987, CI 0.956 to 1.018, *P* = 0.399 for 1-year; RR: 0.978, CI 0.936 to 1.022, *P* = 0.327 for 3-year; RR:0.892, CI 0.783 to 1.015, *P* = 0.084 for 5-year) or mandible (RR: 1.039, CI 0.998 to 1.083, *P* = 0.063 for 1-year; RR: 1.026, CI 0.966 to 1.091, *P* = 0.404 for 3-year; RR: 0.918, CI 0.824 to 1.023, *P* = 0.122 for 5-year) at different defined follow up, respectively (Fig. 3a–c). Furthermore, the arch had a significant impact on the risk ratio difference throughout different follow up periods (*P* < 0.05), while no influence was found when the risk ratio difference of defined follow up (1-, 3-, 5-year independently) was evaluated (*P* = 0.062, *P* = 0.310, *P* = 0.897, respectively). Finally, subgroup analysis (of only the maxilla/mandible independently) in the eight articles^12,40,57–59,61–63,68^ was impractical to conduct since the combination data of both jaws.

**Figure 4:**
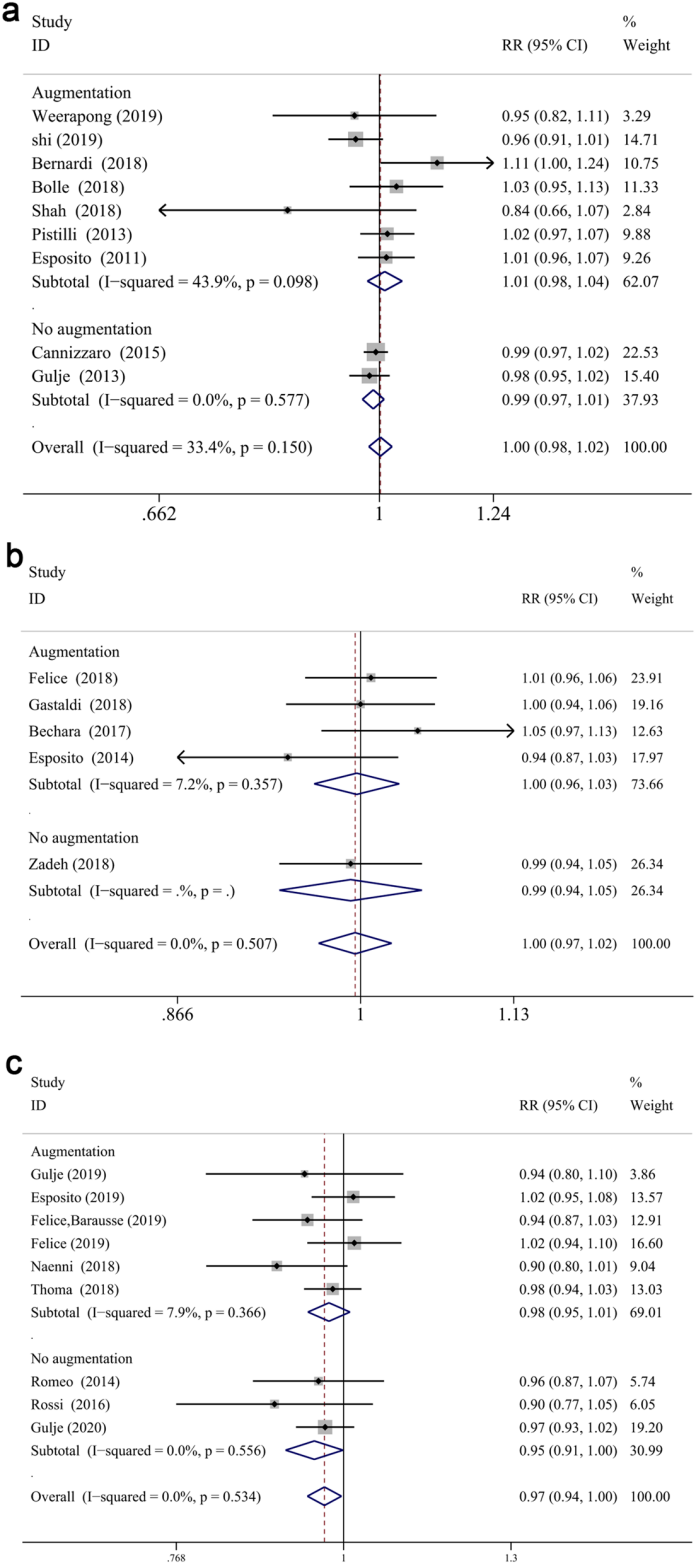
Subgroup analyses for the effects of bone augmentation on survival rate at 1-year (**a**), 3-years (**b**), 5-years (**c**) follow-up. Mantel–Haenszel (MH)-weighted RR < 1 indicated a lower survival rate of extra-short implants than the longer implants.

For further analysis, the influence of augmentation procedure was evaluated. The RRs for reconstructed bone up to 1-year, 3-years and 5-years follow-up were 1.010 (CI 0.978 to 1.044, *P* = 0.542), 0.997 (CI 0.964 to 1.031, *P* = 0.861), 0.977 (CI 0.945 to 1.010, *P* = 0.171), respectively (Fig. 4a–c). And the RRs for native bone up to 1-year, 3-years and 5-years follow-up were 0.989 (CI 0.969 to 1.009, *P* = 0.270), 0.992 (CI 0.938 to 1.049, *P* = 0.786), 0.955 (CI 0.912 to 0.999, *P* < 0.05), respectively (Fig. 4a–c). The subgroup analysis displayed that the survival differences between two groups did not vary significantly when an augmentation procedure was performed or not, while the survival rate of longer implants was higher than that of extra-short implants in native bone after 5-years measurement. Moreover, augment procedure did not impact the results in different defined follow up periods (*P* = 0.228 for 1-year, *P* = 0.933 for 3-year, *P* = 0.436 for 5-year, respectively).

**Figure 5:**
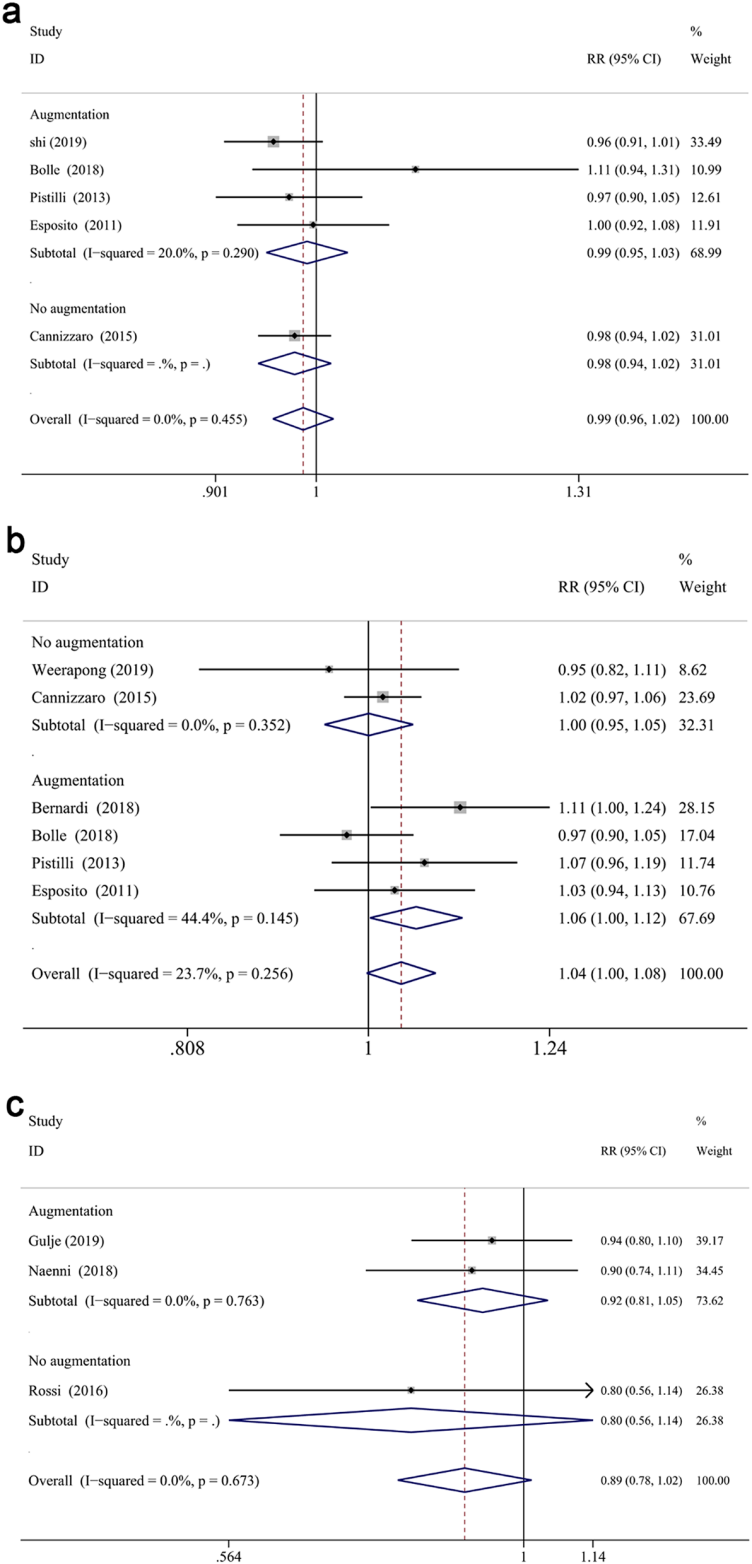
Subgroup analyses for the effects of augmentation on survival rate in the maxilla (**a**) and mandible (**b**) after 1-year measurement, maxilla (**c**) after 5-year measurement. Mantel–Haenszel (MH)-weighted RR < 1 indicated a lower survival rate of extra- short implants than the longer implants.

In investigations with mandibular implants in reconstructed bone^46,48,49,54^, the longer implants showed higher survival rate (RR: 1.058, CI 1.002 to 1.117, *P* < 0.05) than extra-short implants at 1-year follow-up. However, the survival rate of extra-short and longer implants was not significantly different when bone augmentation was not produced in the mandible at 1-year (RR: 1.000, CI 0.949 to 1.054, *P* = 0.988) (Fig. 5b). Similarly, there was no significant difference between two groups at the first year and fifth year in the maxilla with or without bone augmentation procedures (RR: 0.991, CI 0.950 to 1.033, *P* = 0.659 for 1-year reconstructed maxilla; RR: 0.978, CI 0.942 to 1.015, *P* = 0.244 for 1-year native maxilla; RR: 0.923, CI 0.813 to 1.048, *P* = 0.218 for 5-year reconstructed maxilla; RR: 0.804, CI 0.564 to 1.144, *P* = 0.225 for 5-year native maxilla) (Fig. 5a,c). The influence of the bone augmentation procedure at 3-years follow up was not attainable in either maxilla or mandible, since all the studies which reported the survival rate of upper or lower jaw independently underwent augment procedures.

In addition, the RRs of implant survival rate at patient level were calculated by fixed-effects model since the heterogeneity was proved to be low (*P* > 0.05, *I*^2^ = 0%). The RR for overall survival rate between two groups was 0.975 (CI 0.946 to 1.005, *P* = 0.101). Similar with the results of implant level, no significant difference was found at different follow up (RR = 0.979, CI 0.945 to 1.015, *P* = 0.25 at 1-year; RR = 0.995, CI 0.941 to 1.053, *P* = 0.872 at 3-year; RR = 0.956, CI 0.899 to 1.016, *P* = 0.145 at 5-year) (Supplementary Figure 1).

Meta-regression analyses of the RRs for survival rate were performed and results showed that categorical moderators, such as short/long smoking ratio, total smoking percentage, loading time, test of initial stability, were not in significant association with the survival rate differences between two group (*P* > 0.05) at 1-, 3- or 5-year recalls.

**Figure 6:**
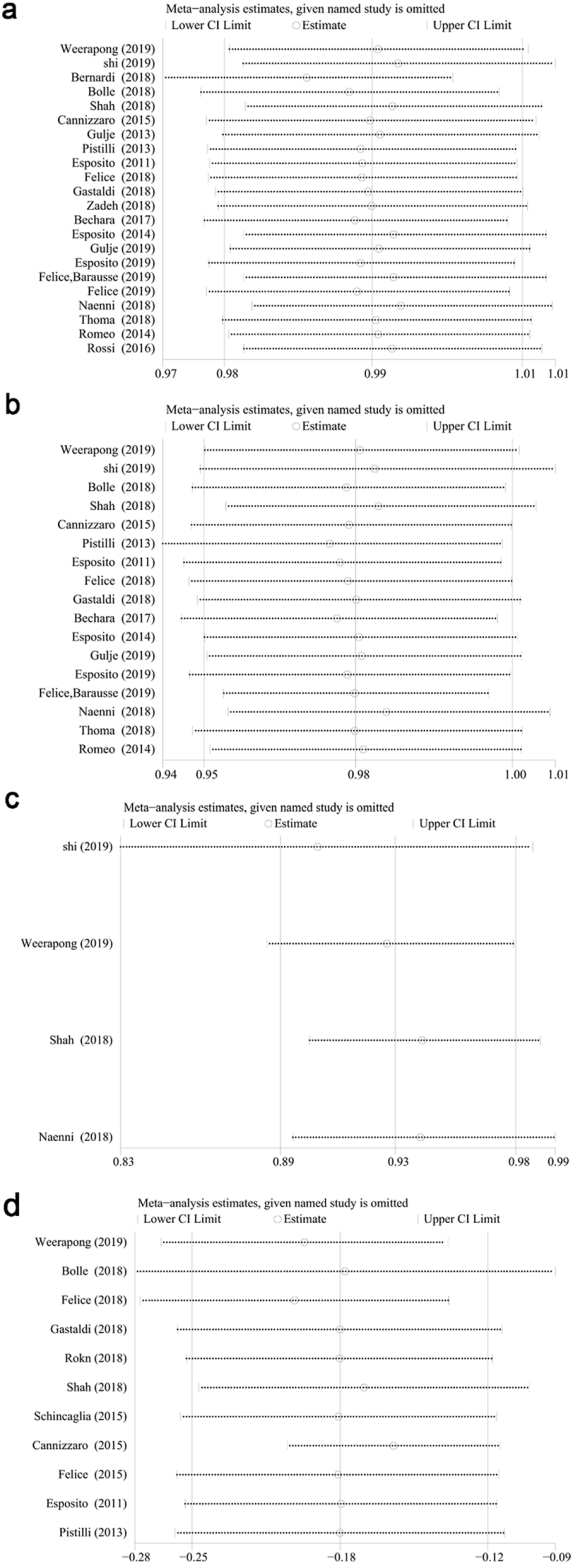
One-out remove graph in survival rate at implant (**a**) and patient level (**b**). One-out remove graph in survival rate (studies with adjustment for within-patient correlation) (**c**) and WMD of MBLs from implant placement at 1-year (**d**).

The sensitivity analyses for all studies in implant and patient level were performed respectively, as well as the studies with correct statistical analyses (Fig. 6a–c). Figure 6a showed that the exclusion of Bernardi et al.^54^ seemed to result in a relatively different meta-analytic estimate. Nevertheless, this difference was insignificant. Accordingly, the combination of investigations was not influenced by a particular one. Similarly, the combination was not influenced by a particular one (Fig. 6b,c).

### Marginal bone loss

Peri-implant marginal bone loss were measured from different baseline between studies. Only studies reporting the outcomes of MBL in patient-level would be analyzed in the present review. 16 articles utilized the baseline measured at the time of implant placement^12,37–40,42–44,46–49,52,61,62,69^, two of which also reported the bone loss from the baseline at prosthetic loading^36,38^. In contrast, nine articles^41,45,53,56,58–60,66,70^ considered the time of prosthetic restoration as baseline exclusively. The data of marginal bone loss was absent in one included study^54^. Therefore, the between-group comparison of marginal bone loss was performed depending on these two different baseline criteria.

#### Marginal bone loss measured from implant placement

**Figure 7:**
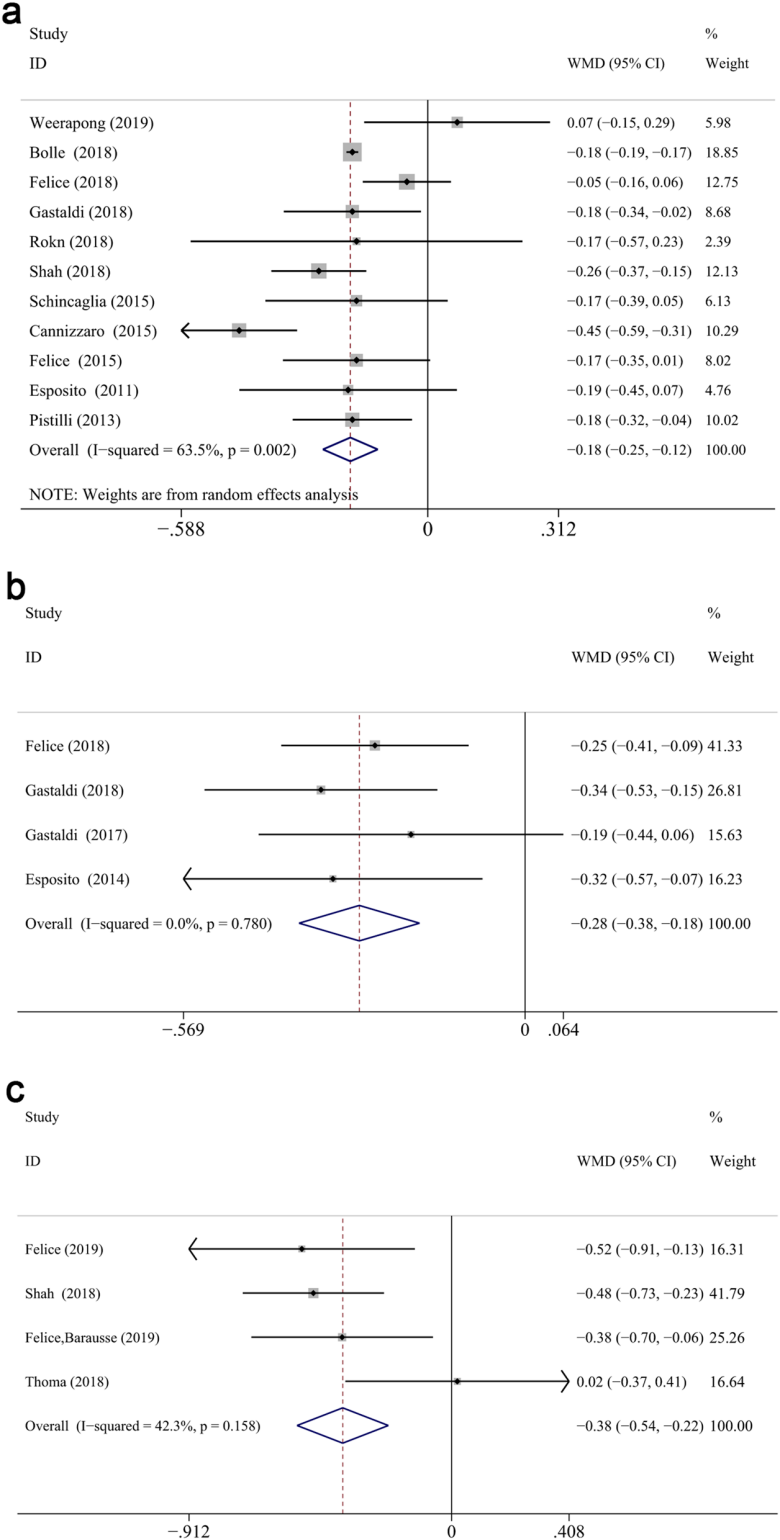
Forest plots (Difference in means) for marginal bone loss, with baseline at implant placement, comparing extra-short and longer implant groups at 1 (**a**), 3 (**b**) and 5 (**c**) years. Negative value in difference in means indicates more MBL in the longer implant group.

**Figure 8:**
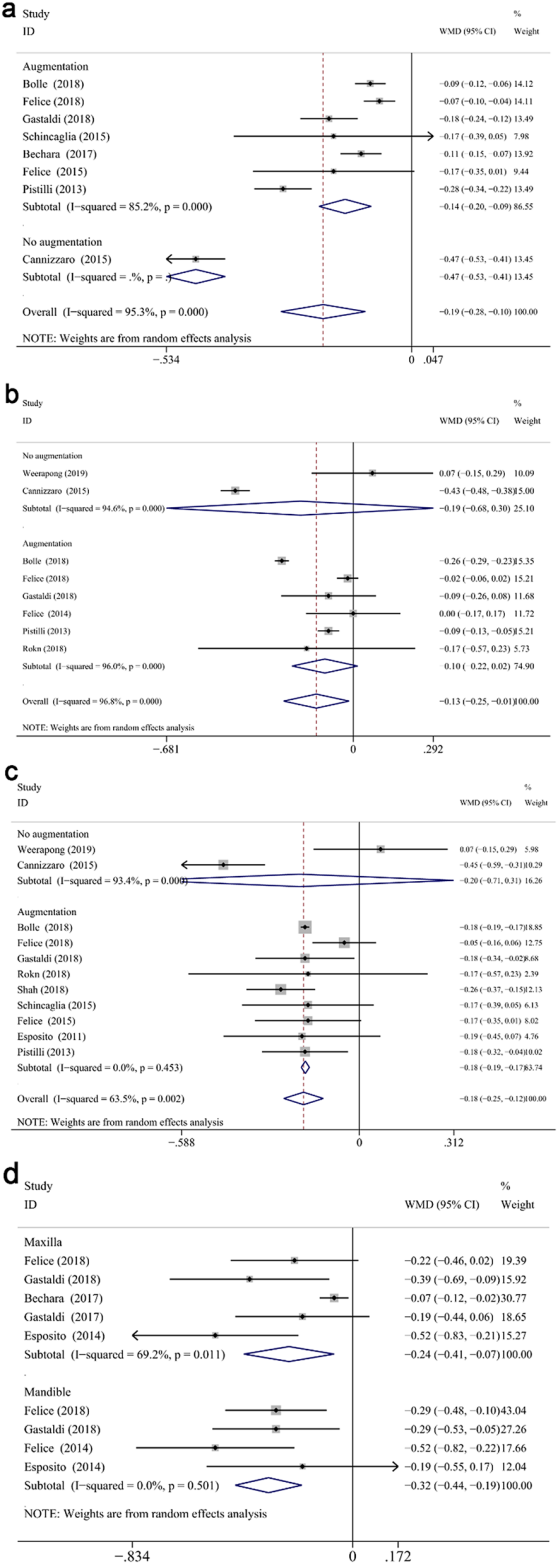
Subgroup analyses for the effects of augmentation on marginal bone loss, with baseline at implant placement, in maxilla (**a**), mandible (**b**) and both jaws (**c**) at 1-year. Subgroup analyses of maxilla/mandible on MBL (from IP) at 3-years follow-up (**d**). Negative value in difference in means indicates more MBL in the longer implant group.

First, extra-short implants showed significantly less MBL measured from IP at different follow up compared with longer implants (WMD: − 0.185, CI − 0.25 to − 0.119, *P* < 0.01 at 1 year; WMD: − 0.276, CI − 0.376 to − 0.176, *P* < 0.01 at 3 years; WMD: − 0.378, CI − 0.536 to − 0.22, *P* < 0.01 at 5 years) (Fig. 7). After 1-year measurement, a significantly less MBL was displayed in the test group in the maxilla (WMD: − 0.193, CI − 0.285 to − 0.101, *P* < 0.001) and mandible (WMD: − 0.134, CI − 0.254 to − 0.015, *P* < 0.05), respectively (Fig. 8a,b). Similarly, MBL of the control group was greater in the maxilla (WMD: − 0.241, CI − 0.412 to − 0.071, *P* < 0.05) and mandible (WMD: − 0.319, CI − 0.444 to − 0.193, *P* < 0.001) (Fig. 8d). In addition, the arch did not impact the mean difference between two groups at 1- or 3-years follow up (*P* = 0.477 for 1-year, *P* = 0.365 for 3-years). Among the 4 included 5-years studies considering the time of implant placement as baseline^12,40,61,62^, only one of them reported the results of maxillary implants^40^, while the others combined the data of both jaws. Therefore, the subgroup analysis (maxilla/mandible) of 5-years was impossible to perform.

When it comes to the influence of augmentation procedure, subgroup analyses demonstrated that in favor of the extra-short implants was found in the reconstructed group at 1 year (WMD: − 0.179, CI − 0.192 to − 0.166, *P* < 0.001), while its native bone counterpart failed to attain significance (WMD: − 0.198, CI − 0.707 to 0.312, *P* = 0.447) (Fig. 8c). Furthermore, subgroup analysis of 1-year follow-up at reconstructed (WMD: − 0.145, CI − 0.202 to − 0.088, *P* < 0.001) and native maxilla (WMD: − 0.470, CI − 0.534 to − 0.406, *P* < 0.001) showed significant difference between two groups (Fig. 8a). While, no significant difference showed in mandible (WMD: − 0.103, CI − 0.223 to 0.016, *P* = 0.090 for reconstructed bone; WMD: − 0.192, CI − 0.681 to 0.297, *P* = 0.442 for native bone) (Fig. 8b). A significant effect of augmentation procedure on the mean difference of maxillary MBL at 1 year was found (coefficient: 0.324, *P* < 0.05). The influence of bone augmentation at 3 and 5 years in both jaws was impossible to be analyzed since all the included studies performed augmentation.

The comparison between extra-short and standard length implants following immediate loading protocol was reported in two included studies at 1-year follow up^47,52^. The loading method (convention/immediate) was demonstrated to significantly relate to the mean difference of maxillary MBL at 1 year through meta-regression analysis (coefficient: 0.324, *P* < 0.01); however, non-significant correlation was found in its mandibular counterpart (*P* = 0.437). Subgroup analyses for the effects of loading method (immediate/conventional) on MBL from IP at 1-year follow up showed significant difference between two groups on maxilla (WMD: − 0.470, CI − 0.534 to − 4.06, *P* < 0.001 for immediate loading; WMD: − 0.145, CI − 0.202 to − 0.088, *P* < 0.001 for conventional loading). Other categorical moderators, such as short/long group smoking ratio, total smoking percentage, test of initial stability, inclusion of heavy smokers, had non-significant association with the MBL differences between groups (*P* > 0.05) at different defined follow up recalls. Interestingly, the total smoking percentage had a positive correlation to MBL difference between two groups at 5-year follow up (coefficient: 1.646,* P* = 0.16) (Fig. 13a), despite the *P* value with no statistical significance. It indicates that higher the number of smokers the higher the tendency of greater MBL occurring in extra-short compared to longer implants.

Random-effect model and subgroup analysis were performed as above since the heterogeneity in WMD of MBLs at 1-year was high (*I*^2^ = 63.5%, CI 30.16% to 80.93%, *P* < 0.05). In addition, the one-out remove method with metaninf module was conducted for the sensitivity analysis, and the exclusion of Cannizzaro-2015^47^ study seemed to result in a relatively different meta-analytic estimate. Nevertheless, this difference was insignificant. Accordingly, the combination of investigations was not influenced by a particular one (Fig. 6d).

#### Marginal bone loss measured from prosthesis restoration

**Figure 9:**
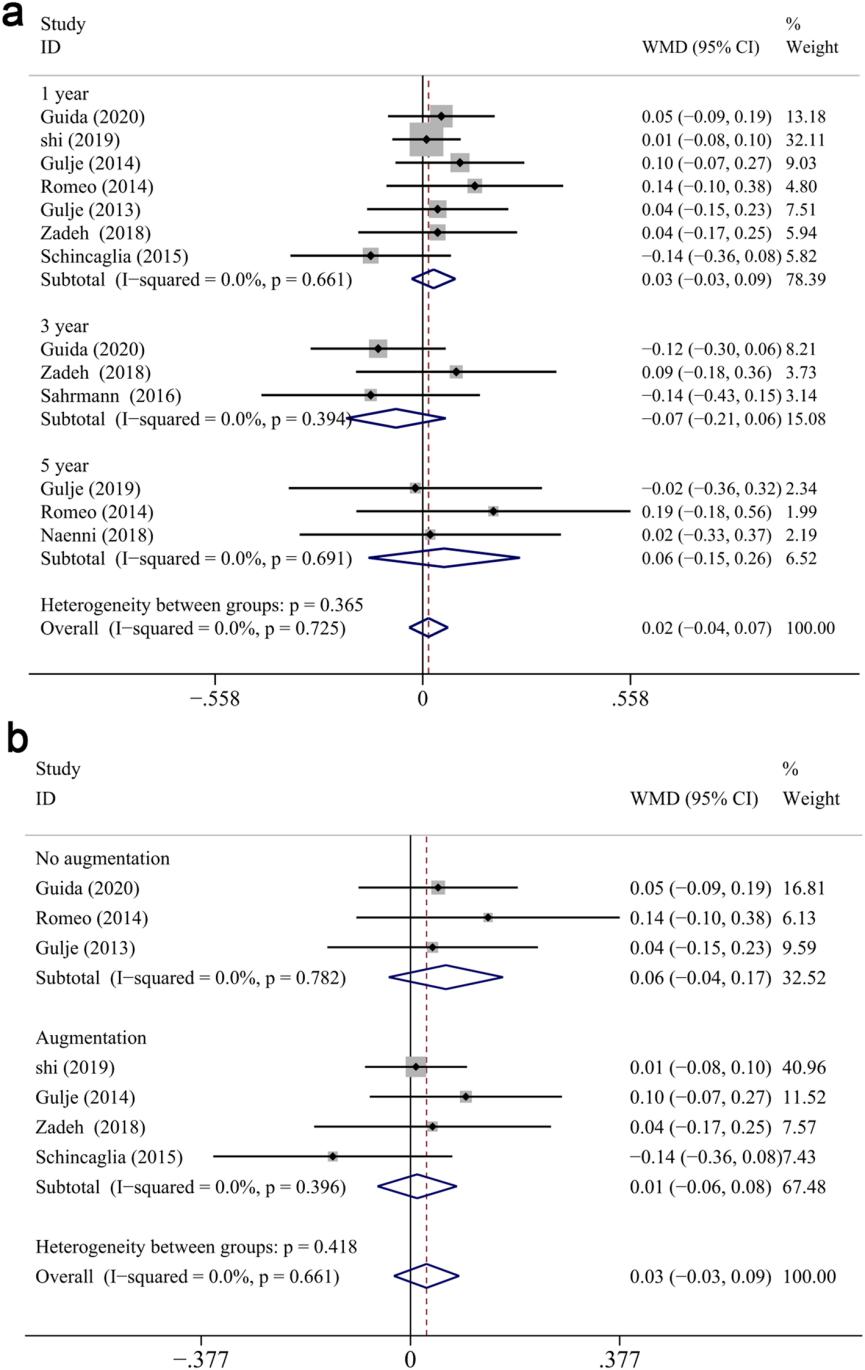
Forest plots (Difference in means) of the marginal bone loss with baseline at prosthesis restoration comparing extra-short with longer implants group at 1-year, 3-year and 5-year follow-up (**a**). Subgroup analyses for effects of augmentation for MBL (from PR) after 1-year measurement (**b**). Negative value in difference in means indicates more MBL in the longer implant group.

There was no significant difference of bone loss measured from PR between groups at different follow up ((WMD: 0.016, CI − 0.036 to 0.068, *P* = 0.555 overall, WMD: 0.029, CI − 0.03 to 0.088, *P* = 0.332 at 1 year; WMD: − 0.072, CI − 0.206 to 0.062, *P* = 0.291 at 3 years; WMD: 0.058, CI − 0.146 to 0.261, *P* = 0.579 at 5-years) (Fig. 9a). Subgroup analysis of the reconstructed/native bone was performed at 1-year, no statistically significant difference displayed between two groups (reconstructed bone: WMD: 0.012, CI − 0.059 to 0.084, *P* = 0.738; native bone: WMD: 0.064, CI − 0.039 to 0.167, *P* = 0.223, respectively) (Fig. 9b). Non-significant association was found between categorical moderators and the mean differences at 1-year (*P* > 0.05). Furthermore, meta-regression analysis could not be performed at 3 or 5 years due to the limit number of included RCTs (3 for 3-years and 3 for 5-years).

### Biological/prosthesis complication rate

**Figure 10:**
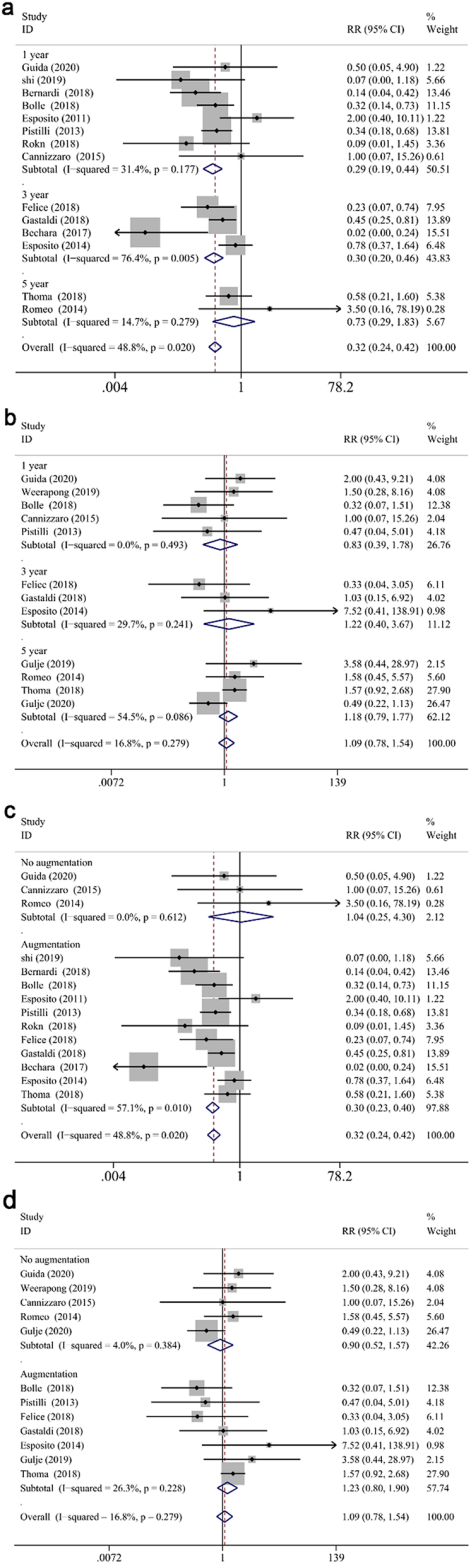
Forest plots (RR) of the biological (**a**) and prosthesis (**b**) complication rate comparing extra-short with longer implants group at 1-year, 3-years and 5-years follow-up. Subgroup analyses for the effects of augmentation on biological (c) and prosthesis (**d**) complication rate in both jaws. Mantel–Haenszel (MH)-weighted RR < 1 indicated a lower complication rate of extra-short implants than the longer implants.

Overall, a significantly less biological complication rate was observed in test group (RR: 0.321, CI 0.243 to 0.422, *P* < 0.001) (Fig. 10a). Nevertheless, the prosthetic complications rate failed to reach statistically significance (RR: 1.092, CI 0.777 to 1.535, *P* = 0.611) (Fig. 10b).

#### Biological complication rate

**Figure 11:**
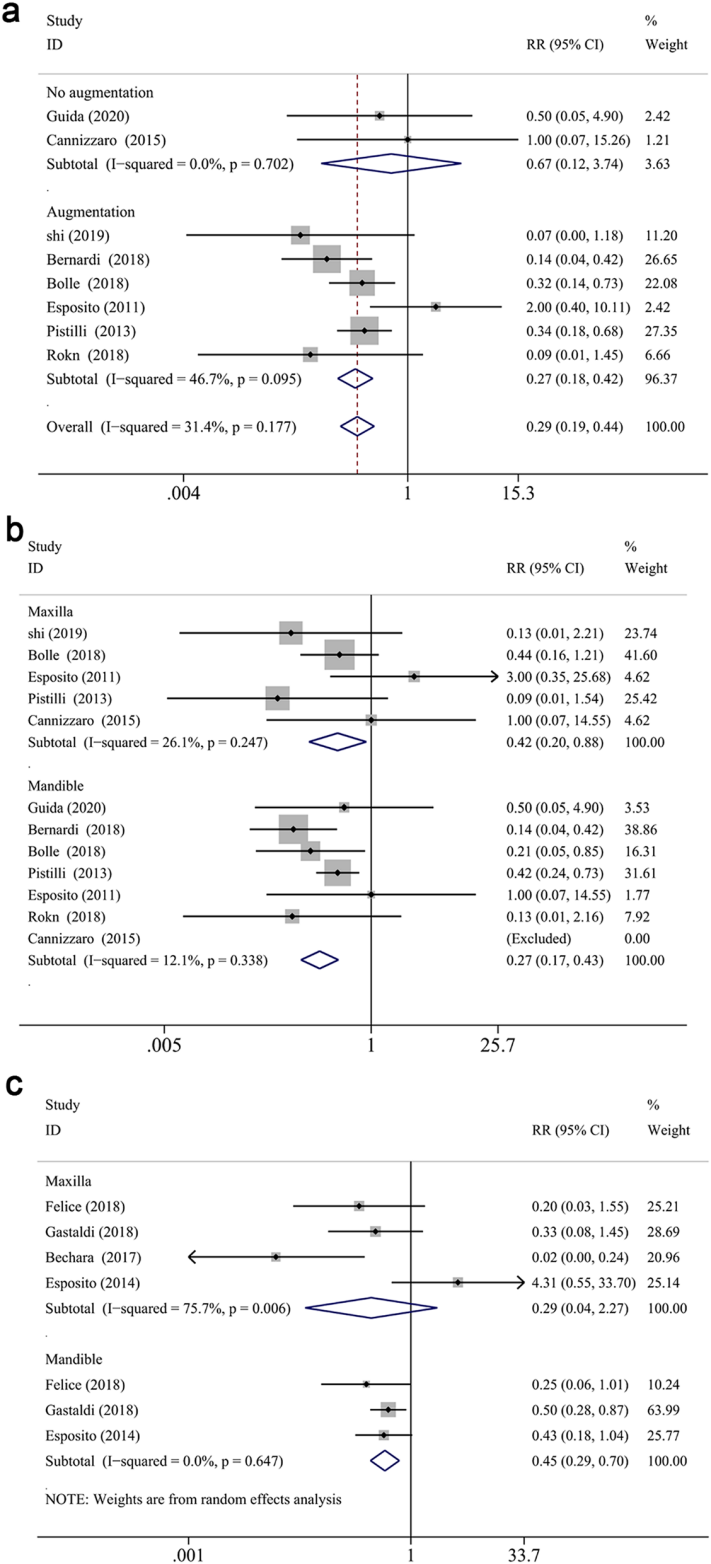
Subgroup analyses for the effects of augmentation on biological complication rate at 1-year (**a**). Subgroup analyses of maxilla/mandible on biological complication rate at 1-year (**b**) and 3-years (**c**) follow-up. Mantel–Haenszel (MH)-weighted RR < 1 indicated a lower complication rate of extra-short implants than the longer implants.

At 1- and 3-years follow-up, the biological complication rate of test group was significantly less than that of control group (RR: 0.289, CI 0.191 to 0.438, *P* < 0.001 at 1-year; RR: 0.304, CI 0.203 to 0.456, *P* < 0.001 at 3-years), while no significant difference was attained at 5 years (RR: 0.726, CI 0.243 to 0.422, *P* = 0.498) (Fig. 10a). Overall, significantly lower biological complication rate was reported in test group in reconstructed bone (RR: 0.305, CI 0.230 to 0.405, *P* < 0.001), while no significant difference was noted in native bone (RR: 1.044, CI 0.254 to 4.298, *P* = 0.952) (Fig. 10c). By subgroup analysis at 1-year, a significantly less biological complication rate was found in test group in both maxilla and mandible (RR: 0.423, CI 0.203 to 0.881, *P* < 0.05; RR: 0.267, CI 0.165 to 0.431, *P* < 0.001, respectively) (Fig. 11b), as well as augmented bone (RR: 0.275, CI 0.179 to 0.423, *P* < 0.001) (Fig. 11a). At 3 years follow up, test group displayed less complication rate in the mandible (RR: 0.446, CI 0.285 to 0.696, *P* < 0.001), while no difference was observed for the maxillary counterpart (RR: 0.291, CI 0.037 to 2.267, *P* = 0.239) (Fig. 11c). A subgroup meta-analysis of 5-year follow-up was impossible to perform due to the only two investigation that reported biological complications after 5 years measurement^40,58^. Furthermore, meta-regression analysis demonstrated that neither location nor augmentation procedure impact the between-groups comparisons (*P* = 0.750, *P* = 0.787, respectively). Other categorical moderators, such as total smoking percentage (*P* = 0.898), S/L ratio (*P* = 0.758), inclusion of heavy smokers (*P* = 0.758), were not significantly correlated with the differences of biological complications in between-group comparison.

#### Prothesis complication rate

**Figure 12:**
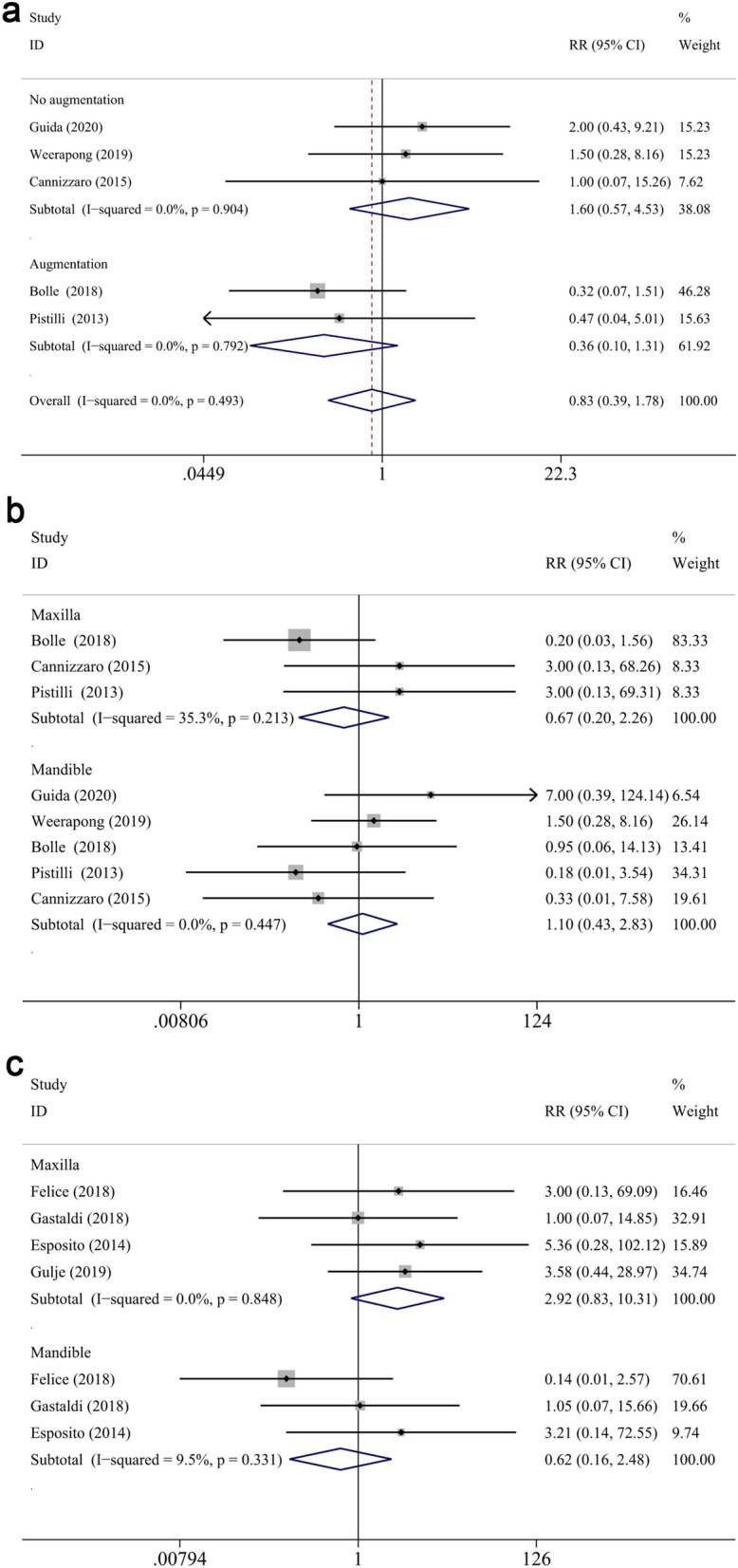
Subgroup analyses for the effects of augmentation on prosthesis complication rate at 1-year (**a**). Subgroup analyses of maxilla/mandible on prosthesis complication rate at 1-year (**b**) and 3-years (**c**) follow-up. Mantel–Haenszel (MH)-weighted RR < 1 indicated a lower complication rate of extra-short implants than the longer implants.

Non-significant difference of prothesis complication rate was found between groups (RR: 0.834, CI 0.390 to 1.781, *P* = 0.639 at 1 year; RR: 1.219, CI 0.405 to 3.673, *P* = 0.725 at 3 years, RR: 1.181, CI 0.790 to 1.767, *P* = 0.418 at 5 years, respectively). Overall, no significant difference was noted in subgroup analyses of constructed/native bone between groups (RR: 1.230, CI 0.798 to 1.897, *P* = 0.349 for reconstructed bone; RR: 1.591, CI 0.710 to 3.568, *P* = 0.259 for native bone) (Fig. 10d). Similarly, subgroup analysis according to maxilla/mandible and reconstructive/native bone also showed no significant difference between groups at 1 or 3 years follow up, respectively (RR: 0.667, CI 0.196 to 2.263, *P* = 0.516 for maxilla at 1 year; RR: 1.104, CI 0.431 to 2.831, *P* = 0.836 for mandible at 1 year; RR: 2.917, CI 0.825 to 10.314, *P* = 0.097 for maxilla at 3 year; RR: 0.621, CI 0.156 to 2.475, *P* = 0.499 for mandible at 3 year; RR: 0.363, CI 0.100 to 1.310, *P* = 0.122 for reconstructed bone at 1 year; RR: 1.600, CI 0.565 to 4.528, *P* = 0.376 for native bone at 1 year) (Fig. 12a–c). Meanwhile, in spite of no statistical significance, the RR > 1 in native bone, while RR < 1 in reconstructed bone. It implies that the prosthesis complication rate tends to be higher in test group in native bone, and reconstructed bone subgroup had inverse pattern, preferring test group.

**Figure 13:**
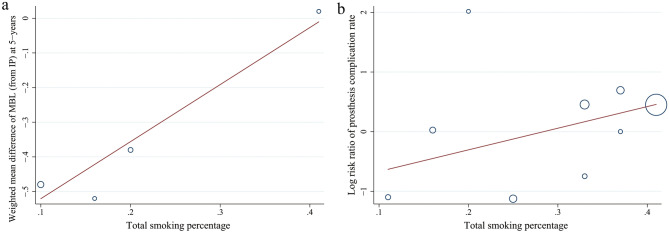
Meta-regression analyses. (**a**) Total smoking percentage had a positive correlation to the difference in mean of 5-year MBL from implant placement between two groups without statistical significance (coefficient: 2.787; p = 0.506); τ^2^ = 0.196; I^2^ = 87.45%; Adj R^2^ = − 12.57%. (**b**) Total smoking percentage had a positive correlation to the RRs for prosthesis complication rates between two groups without statistical significance (coefficient: 2.937; p = 0.570); τ^2^ = 0.000; I^2^ = 4.400%.

Meta-regression was performed and no covariate was found statistically associated with RR for prothesis complication rate between two groups throughout different follow up periods (*P* > 0.5). Nevertheless, despite no statistically significant (*P* = 0.192), a positive correlation between total smoking percentage and prothesis complications in all studies was found (coefficient = 3.628, Fig. 13b). Hence, it was indicated that the more smokers were included the higher the trend of prothesis complications appearing in test group.

## Discussion

To the best of our knowledge, this is the first systematic review comparing the survival rate, marginal bone loss, biological and prosthesis complication rate between extra-short implants (≤ 6 mm) and longer implants (≥ 8 mm) at both jaws, maxilla or mandible independently, with and without bone augmentation procedures.

### Survival rate

The survival rate of extra-short implants was found comparable to the longer implants at 1- and 3-years follow-up in the present review, while significantly higher survival rate was found in longer group at 5-year. The present outcomes resembled the reports of previous meta-analyses, in which the survival rate of short implants in long-term follow-up was lower than long implants^14,29^, and more updated RCTs were included in the present meta-analysis compared to preceding. Moreover, moderate consistent findings across studies were corroborated by the relative deficiency of heterogeneity (*I*^2^ = 17.8%, CI 0 to 53.0%, *P* = 0.241) and the limited dispersion of the funnel plot, suggesting relatively low between‐study heterogeneity. According to the GRADE system, pooling of studies on implants survival rate provided moderate-quality evidence.

For subgroup analyses, no significant difference between test and control groups was found when considering the influence of implant position (maxilla/mandible). In spite of this, the survival rates of the extra-short implants displayed a more serious downward trend over time than longer implants both in upper and lower jaws, which may imply the not optimistic long-term (more than 5 years) clinical outcomes. Interestingly, in favor of the longer implants was found in the native bone group at 5 year, while its reconstructed counterpart failed to attain significance, indicating the extra-short implants could be an acceptable alternative to longer implants in atrophic posterior arch. Meanwhile, a better result for extra-short implants could be anticipated as the development of implant surface modification and shape design^71,72^. Interestingly, mandibular longer implants with vertical bone augmentation displayed a slightly less survival rate than the extra-short implants at 1 year, while the survival rate of extra-short implants was no better than that of mandibular long implants without augmentation. Although the rapid development and wide application of vertical bone augmentation, the high technique-sensitivity and possible complications could remain the essential stimulus of implant failure^73^, which may contribute to the above outcomes.

Information of multiple sites within a single subject are frequently collected in oral health study designs, introducing generally positive correlation among responses within subject^74^. Much less attention has been devoted to this essential issue. In fact, however, increased risk of bias, leading to overestimation of performance of implants, or inappropriate conclusions drawing from the outcomes may be attributed to this substantial problem^75^. Among the 31 included RCTs, 4 of them assigned one implant per patient, in which the within-patient correlation did not need to take into consideration. When it comes to the rest of these RCTs with multiple implants each patient, only one study^53^ randomly selected one implant per patient, in which the within-patient correlation could be avoided to some extent. Nevertheless, none of the remaining 25 RCTs adjusted for within-patient correlation. Almost all of these RCTs just reported the number of survived/total implants in contingency table, and compared the frequency of survived implants between two groups by statistical significance test (eg. Fisher’s exact test). Thus, the result of survival rate, not only in the present systematic review but also probably all the previous review on this topic, should be interpreted with caution, since it is particularly hard for subsequent meta-analysis based on these RCTs to calculate the adjusted RR estimate.

Furthermore, even though the only RCT attempted to deal with the within-patient correlation by randomly selecting one implant per participant, it is an inefficient design due to the loss of potentially highly valuable information and inferior statistically valid standard error estimate^76^. The simplest strategy to avoid this problem, which is also the majority of the included RCTs employed, is to generate a summary statistic over all implants in the same patient, and then the standard statistical methods for independent observations could be applied. However, as the result, the sensitivity to effects, power and precision may be decreased due to the averaging over many sites and reduced sample size^74^. The RR estimation (at patient level) of the studies in which patient was considered as analysis unit was calculated so as to avoid the influence of within-patient correlation on pooled RR as much as possible. And the results seemed to be similar to those at implant level.

### Marginal bone loss

MBL calculated and compared at the patient level, instead of the implant level, were analyzed in this systematic review. The MBL from IP showed that the bone resorption for test group was less than control group with the statistical significance. And as the time goes on, there was greater mean difference of the MBL between two groups, which indicated that, in terms of MBL, extra-short implants would prior to longer implants especially in long-term prognosis. Nevertheless, when the length of implant was taken into consideration, the greater absolute value of MBL may not exactly equal to the greater bone loss relative to the length of implant. In other words, greater MBL of longer implants compared to extra-short implants may not absolutely leads to the greater C/I ratio of longer implants. Moreover, bone augmentation procedure was performed in all the included 3- and 5-years RCTs, which may contribute to the greater MBL in control group. In addition, when it comes to the subgroup analyses for the effect of augmentation, the MBL from IP at both jaws of extra-short implants was statistically significantly less than longer implants in reconstructed bone at 1 year, while insignificant difference was found in native bone, which corroborated the effect of bone augmentation on MBL.

Similar results were found in subgroup analysis of implant position (maxilla/mandible) at 1- and 3-years follow-up. And significant difference was found in maxilla with and without augmentation at 1 year. Surprisingly, mean differences of MBL with augmentation was less than that without augmentation, which was contrary to the above outcomes. Nevertheless, this result, which was speculated from the analyses of only one investigation^47^ installing long implants without bone augmentation and seven articles^38,42–44,46,48,55^ utilizing augmentation procedure (sinus lift) when indicated. Moreover, that investigation^47^ was the only study that employed the immediately loaded implants among all the included 1-year studies on maxilla. In present analysis, the loading method (immediate/conventional) had a significant impact on the mean difference of MBL at maxilla after 1-year measurement, and greater mean difference was found in the immediately loaded implants. In addition, significant resorption was prone to occur after vertical bone augmentation, particularly in the mandible^77^, and higher MBL after augmentation was validated in the previous meta-analyses^17,25,78^. Therefore, the immediate loading was likely to bear the mainly responsibility for the greater mean difference of MBL between two groups in this study. The heterogeneity in the MBL measured from IP at 1 year was relatively severe, and the potential sources of heterogeneity would be augmentation procedure, implants location and loading method. While, based on the sensitivity analysis and publication bias assessment, this result was credible. In addition, according to the GRADE system, pooling of studies on MBL measured from IP provided low-quality evidence and from PR were rated as moderate-quality.

There was no significant difference of MBL from PR between test and control groups in all defined follow up periods. Besides, the subgroup analyses for the effect of implants position and augmentation procedure showed the similar results. Thus, a higher MBL from IP was observed in control group in this article as above. The bone remodeling process, which was known to proceed along with an adaptive biological width after the second stage of implant surgery and prior to prosthetic loading, could account for the different results of MBL measured from IP and PR^79^. Compared to implant placement, the bone should be more stable when prosthesis loaded and the impact of the initial bone remodeling could be primarily avoided if baseline was measured at this time. In this way, prosthetic factors which may affect the marginal bone resorption could be better analyzed and comprehended. In addition, implant placement without bone augmentation procedure were employed in some of the selected RCTs, as well as relatively small number of analyzed studies, may account for the insignificant difference between two groups.

### Complications

Complications in this systematic review were calculated and compared at the patient level, instead of the implant level. The extra-short implants displayed a significantly lower biological complication rate than longer implants in both maxilla and mandible, especially in reconstructed bone, which was corroborated in the previous meta-analyses^2,24,25,29^. The higher complication rate of longer implants in reconstructed bone, such as paresthesia, graft infection, graft resorptions, perforation of the sinus membrane, could be caused by the augmentation procedures. Moreover, compared to short implant placement, the augmentation procedure is characterized by relatively time-consuming and suffering, which might be slightly related to the high complication rate of long implants with augmentation, as some biological complications were self-reported by patients.

In present meta-analysis, no statistically significant difference of prosthesis complication rate was found between two groups, which was similar with previous studies^15^. Meanwhile, despite no statistical significance, the extra-short implants trended to have higher prosthesis complication rate in native bone, and inverse pattern was observed in reconstructed bone at 1 year. It was also reported that the rate of prosthesis complication in extra-short implants (≤ 6 mm) was higher than long implants (≥ 10 mm)^29^. The difference may be caused by the different definition of control group that the implants of length no less than 8 mm were all considered as control group in this review. The higher C/I ratio is often considered as a risk of extra-implants for prosthesis restoration, but several investigations have failed to demonstrate a detrimental effect of this parameter on the rates of prosthesis complications^10,80–82^. In addition, meta-regression of C/I ratio was hard to accomplish since only four series of studies in included articles reported C/I ratio^40,41,50,57^. Thus, together with our results, the authors suggest that the possible prosthesis complication rate of extra-short implants would be approximately higher than longer implants in long term prognosis.

The Consolidated Standards of Reporting Trials (CONSORT) statement, which was developed in 1996, updated in 2001 and 2010^83–86^ and endorsed by many biomedical journals, aims to improve clarity and consistency of transparency of reporting in RCTs. There was more possibility for RCTs published after 2010 to have superior performance when evaluated with Cochrane Risk of Bias tool in total^87^. However, the use of the CONSORT statement varies among RCTs since only 20 out of 31 included articles adhere to the CONSORT statement, according to the present systematic review. Moreover, only eight out of thirty included RCTs had been registered in a public database and only two of them were prior registered^53,66^, which has been required by many medical journals for years. Therefore, a further encouragement to dental researchers to register in public database and adhere to the CONSORT statement should be established so as to improve the quality of RCTs and consequently better patient care.

Compared to previous meta-analyses^2,29^, more articles reporting a 5-years follow-up were included, together with low publication bias and heterogeneity, leading to a more reliable outcome of long-term follow up. The quality of evidence was assessed using the GRADE approach and the majority were found to be moderate-quality, while the MBL measured from IP showed low-quality due to the imprecision and inconsistency of included RCTs^88,89^. The result of MBL from IP should be interpreted with caution since the low-quality and high heterogeneity. Some subgroup analyses in long-term follow up studies, such as maxilla/mandible for 5-years MBL and complication rate, were impossible to perform since the lack of maxillary implants studies at 5 years. Moreover, the meta-regression analyses of potential associating clinical variants in long-term follow up, suggesting the direction for future research, should be interpreted with caution due to the limited number of 5-years studies reporting analyzable clinical details. And more notably, nearly all of the previous RCTs and systematic reviews on this topic ignored the importance of the statistical issues, especially the problem of within-patient correlation during the analysis of implant survival rate. The comparisons of survival rate at implant level between short and long implants in systematic reviews should be interpreted with caution since the imperfect statistical method of design and analysis in included RCTs.

## Conclusion

The above outcomes indicate that the placement of extra-short implants (≤ 6 mm) is an acceptable alternative to longer implants (≥ 8 mm) with bone augmentation in atrophic posterior arch, due to the comparable survival rate, less bone resorption as well as lower biological complication rate. Further high-quality and prior registered RCTs with a longer follow-up period (at least 5 years), appropriate statistics approaches, satisfactory adherence to CONSORT statement are required to corroborate the present outcomes.

## Supplementary Information

Supplementary Information 1.

Supplementary Information 2.

Supplementary Information 3.
