# Author Correction: A meta-analysis indicating extra-short implants (≤ 6 mm) as an alternative to longer implants (≥ 8 mm) with bone augmentation

**DOI:** 10.1038/s41598-021-98441-7

**Published:** 2021-09-15

**Authors:** Xiaoran Yu, Ruogu Xu, Zhengchuan Zhang, Yang Yang, Feilong Deng

**Affiliations:** 1 Department of Oral Implantology, Hospital of Stomatology, Guanghua School of Stomatology, Sun Yat-Sen University, 56 Ling Yuan Xi Road, Guangzhou, 510006 Guangdong People’s Republic of China; 2 Guangdong Provincial Key Laboratory of Stomatology, 74 Zhong Shan Er Road, Guangzhou, 510006 Guangdong People’s Republic of China

Correction to: *Scientific Reports* 10.1038/s41598-021-87507-1, published online 14 April 2021

The original version of this Article contained an error in Table 2, where the overall evaluation in row “Guida et al. 2020” was incorrect,

“(?) some concerns”.

now reads:

“(+) low risk”.

The original Table 2 and accompanying legend appear below.

**Table 2:**
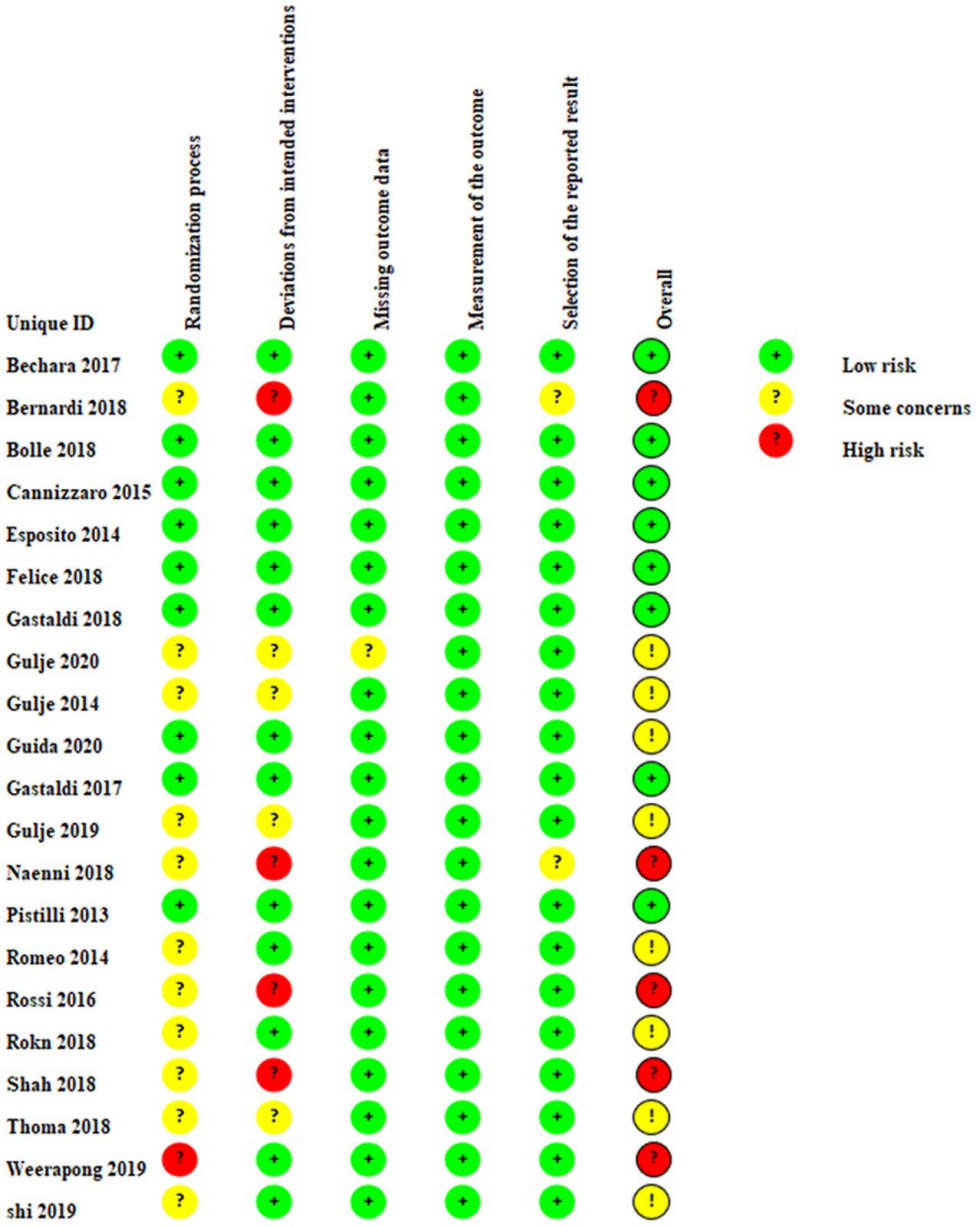
Quality assessment of included studies by ROB 2.

Generated by RevMan Web, https://revman.cochrane.org.

As a result, in the Results section, under subsection ‘Result, Risk of bias and quality of evidence’,

“Five studies were considered as having a high risk of bias, and judgments expressed “some concern” in eight studies, while the remaining were characterized by a low risk of bias.”

now reads:

“Five studies were considered as having a high risk of bias, and judgments expressed “some concern” in seven studies, while the remaining were characterized by a low risk of bias.”

The original Article has been corrected.

