# Supplementary material for: A meta-analysis indicating extra-short implants (≤ 6 mm) as an alternative to longer implants (≥ 8 mm) with bone augmentation
Source: Sci Rep. 2021 Apr 14;11:8152. doi: 10.1038/s41598-021-87507-1 (PMC8047002; doi:10.1038/s41598-021-87507-1)
Supplement: Supplementary file 1 — Supplementary Information 1. [file 41598_2021_87507_MOESM1_ESM.docx]

**Title**

A meta-analysis indicating extra-short implants (≤6mm) as an alternative to longer implants (≥8mm) with bone augmentation

**Authors**

Xiaoran Yu ^a,b,*^, Ruogu Xu ^a,b,*^, Zhengchuan Zhang ^a,b^, Yang Yang ^a,b^, Feilong Deng ^a,b^

^a^ Department of Oral Implantology, Hospital of Stomatology, Guanghua School of Stomatology, Sun Yat-Sen University, 56 Ling Yuan Xi Road, Guangzhou, 510006, Guangdong Province, PR China

^b^ Guangdong Provincial Key Laboratory of Stomatology, 74 Zhong Shan Er Road, Guangzhou, 510006, Guangdong Province, PR China

^*^Xiaoran Yu and Ruogu Xu contributed equally to this work

**Corresponding author**

Feilong Deng ^a,b^, DDS, Professor

^a^ Department of Oral Implantology, Hospital of Stomatology, Guanghua School of Stomatology, Sun Yat-Sen University, 56 Ling Yuan Xi Road, Guangzhou, 510006, Guangdong Province, PR China

^b^ Guangdong Provincial Key Laboratory of Stomatology, 74 Zhong Shan Er Road, Guangzhou, 510006, Guangdong Province, PR China

Email: dengfl@mail.sysu.edu.cn

Tel: +86 20 8386 2537

Fax: +86 20 8382 2807


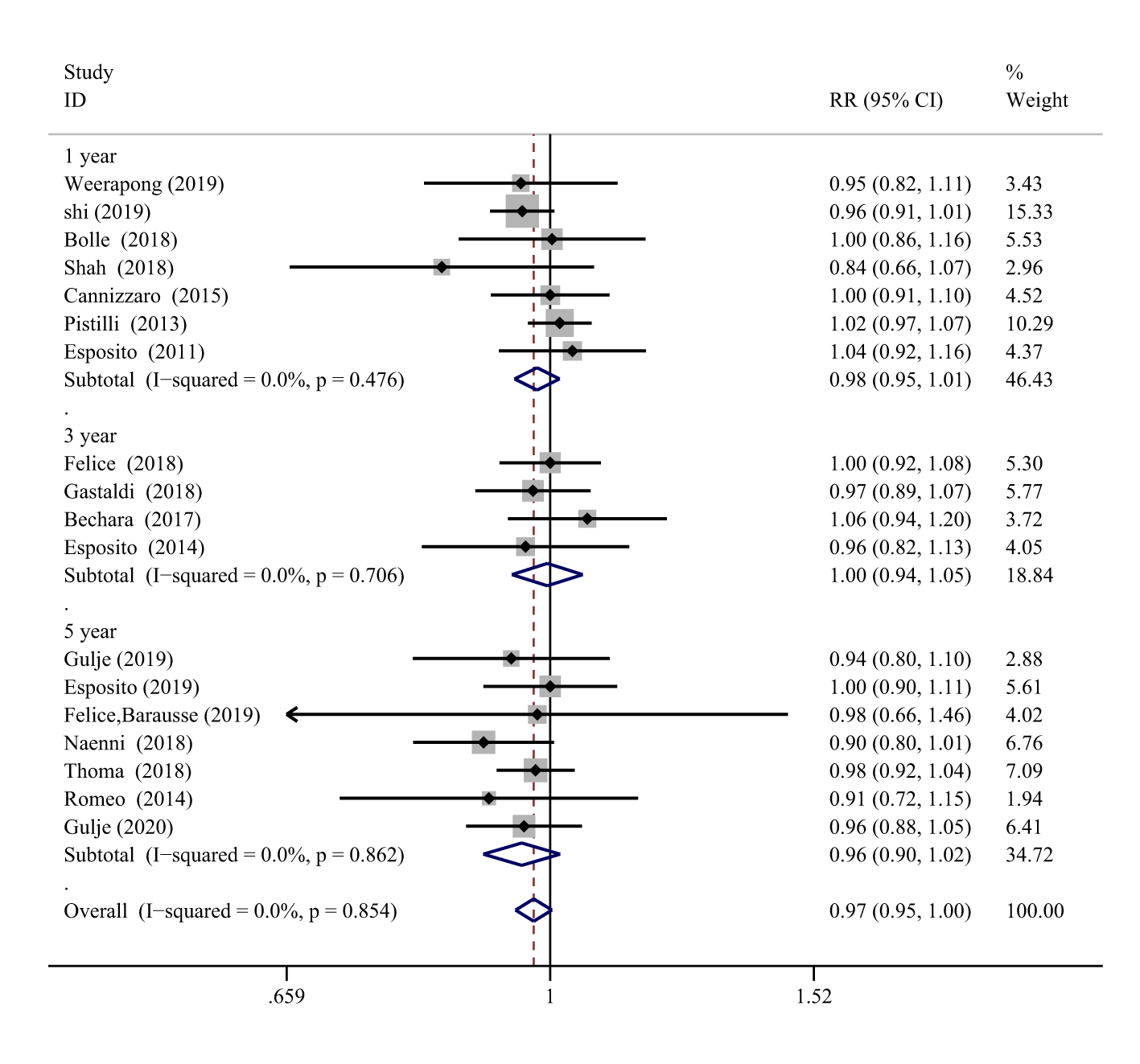


**Supplementary Figure 1** Forest plots (RR) of the survival rate (in patient level) comparing extra-short with longer implants. Mantel–Haenszel (MH)- weighted RR < 1 indicated a lower survival rate of extra-short implants than the longer implants.


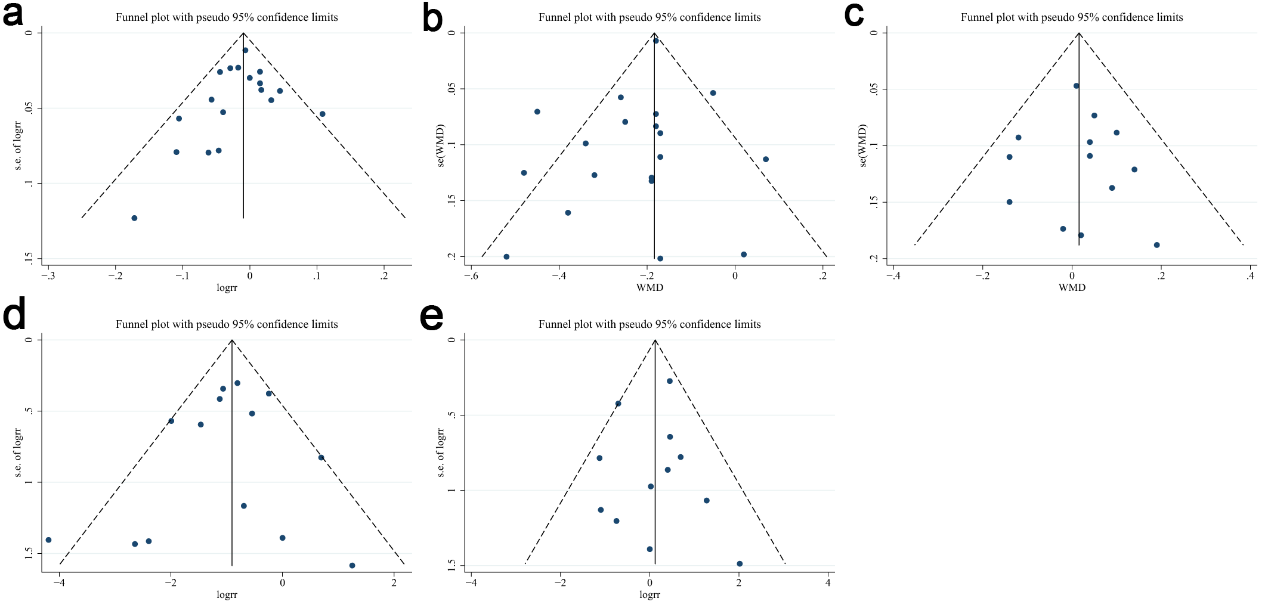


**Supplementary Figure 2** Funnel plot of survival rate (a), MBL from IP (b) and PR (c), biological (d) and prosthesis complication rate (e).


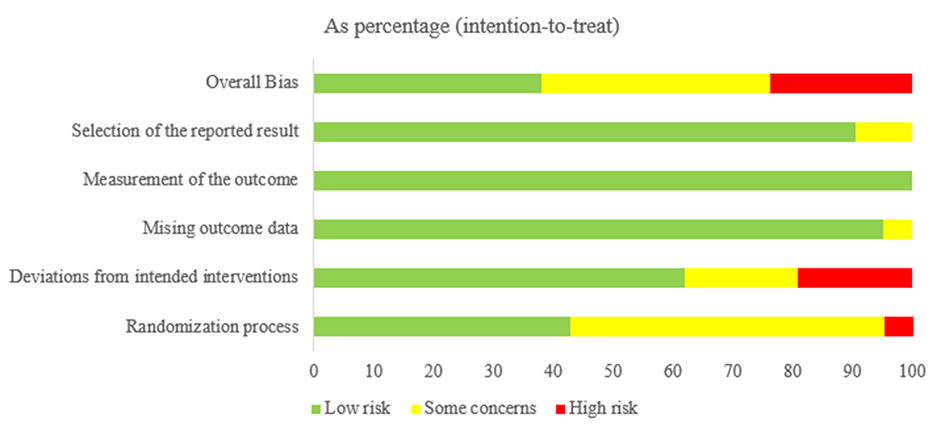


**Supplementary Figure 3** Quality assessment of included studies by ROB 2.
