# Supplementary material for: A meta-analysis indicating extra-short implants (≤ 6 mm) as an alternative to longer implants (≥ 8 mm) with bone augmentation
Source: Sci Rep. 2021 Apr 14;11:8152. doi: 10.1038/s41598-021-87507-1 (PMC8047002; doi:10.1038/s41598-021-87507-1)
Supplement: Supplementary file 2 — Supplementary Information 2. [file 41598_2021_87507_MOESM2_ESM.docx]

**Title**

A meta-analysis indicating extra-short implants (≤6mm) as an alternative to longer implants (≥8mm) with bone augmentation

**Authors**

Xiaoran Yu ^a,b,*^, Ruogu Xu ^a,b,*^, Zhengchuan Zhang ^a,b^, Yang Yang ^a,b^, Feilong Deng ^a,b^

^a^ Department of Oral Implantology, Hospital of Stomatology, Guanghua School of Stomatology, Sun Yat-Sen University, 56 Ling Yuan Xi Road, Guangzhou, 510006, Guangdong Province, PR China

^b^ Guangdong Provincial Key Laboratory of Stomatology, 74 Zhong Shan Er Road, Guangzhou, 510006, Guangdong Province, PR China

^*^Xiaoran Yu and Ruogu Xu contributed equally to this work

**Corresponding author**

Feilong Deng ^a,b^, DDS, Professor

^a^ Department of Oral Implantology, Hospital of Stomatology, Guanghua School of Stomatology, Sun Yat-Sen University, 56 Ling Yuan Xi Road, Guangzhou, 510006, Guangdong Province, PR China

^b^ Guangdong Provincial Key Laboratory of Stomatology, 74 Zhong Shan Er Road, Guangzhou, 510006, Guangdong Province, PR China

Email: dengfl@mail.sysu.edu.cn

Tel: +86 20 8386 2537

Fax: +86 20 8382 2807

38 articles were excluded after full text articles assessment for reasons as follow:

Three articles, Zygogiannis, K., et al., 2018 [1], Sahrmann, P., et al., 2017 [2], Shi, J. Y., et al., 2015 [3] were excluded since there was no explicit report of survival rate.

Twelve were excluded because the short implants investigated in those studies were longer than 6mm (Taschieri, S., et al., 2018 [4], Yu, H., et al., 2017 [5], Mendoza-Azpur, G., et al., 2016 [6], Esposito, M., et al., 2015 [7], Felice, P., et al., 2014 [8], Cannizzaro, G., et al., 2012 [9], Esposito, M., et al., 2011 [10], Felice, P., et al., 2010 [11], Cannizzaro, G., et al., 2009 [12], Cannizzaro, G., et al., 2008 [13], Felice, P., et al., 2009 [14], Zygogiannis, K., et al., 2017 [15]).

Six were excluded due to the short follow-up period (no more than 12 months): Esposito, M., et al., 2015 [16], Esposito, M., et al., 2012 [17], Felice, P., et al., 2011 [18], Zhang, X. M., et al., 2017 [19], Esposito, M., et al., 2016 [20], Felice, P., et al., 2012 [21].

Eleven were excluded since there was no direct comparison between the clinical outcomes of short and longer implants: Hsu, A., et al., 2016 [22], Al-Hashedi, A. A., et al., 2016 [23], Telleman, G., et al., 2014 [24], Telleman, G., et al., 2013 [25], Grunder, U., et al., 1999 [26], Telleman, G., et al., 2017 [27], Ozturk, V. O., et al., 2017 [28], Muller, F., et al., 2013 [29], Schwindling, F. S., et al., 2018 [30], Schwarze, M., et al., 2018 [31], Blanco, J., et al., 2018 [32].

Six were excluded because they were prospective, case report or case series, instead of randomized controlled trials: Nedir, R., et al., 2017 [33], Rossi, F., et al., 2016 [34], Nedir, R., et al., 2016 [35], Bergendal, T., et al., 1998 [36], Gunne, J., et al., 1999 [37], de Molon, R. S., et al., 2017 [38].

1. Zygogiannis, K., I.H. Aartman, and D. Wismeijer, *Implant Mandibular Overdentures Retained by Immediately Loaded Implants: A 1-Year Randomized Trial Comparing Patient-Based Outcomes Between Mini Dental Implants and Standard-Sized Implants.* Int J Oral Maxillofac Implants, 2018. **33**(1): p. 197-205.

2. Sahrmann, P., P. Schoen, N. Naenni, et al., *Peri-implant bone density around implants of different lengths: A 3-year follow-up of a randomized clinical trial.* J Clin Periodontol, 2017. **44**(7): p. 762-768.

3. Shi, J.Y., Y.X. Gu, S.C. Qiao, et al., *Clinical evaluation of short 6-mm implants alone, short 8-mm implants combined with osteotome sinus floor elevation and standard 10-mm implants combined with osteotome sinus floor elevation in posterior maxillae: study protocol for a randomized controlled trial.* Trials, 2015. **16**: p. 324.

4. Taschieri, S., A. Lolato, T. Testori, et al., *Short dental implants as compared to maxillary sinus augmentation procedure for the rehabilitation of edentulous posterior maxilla: Three-year results of a randomized clinical study.* Clin Implant Dent Relat Res, 2018. **20**(1): p. 9-20.

5. Yu, H., X. Wang, and L. Qiu, *Outcomes of 6.5-mm Hydrophilic Implants and Long Implants Placed with Lateral Sinus Floor Elevation in the Atrophic Posterior Maxilla: A Prospective, Randomized Controlled Clinical Comparison.* Clin Implant Dent Relat Res, 2017. **19**(1): p. 111-122.

6. Mendoza-Azpur, G., M. Lau, E. Valdivia, et al., *Assessment of Marginal Peri-implant Bone-Level Short-Length Implants Compared with Standard Implants Supporting Single Crowns in a Controlled Clinical Trial: 12-Month Follow-up.* Int J Periodontics Restorative Dent, 2016. **36**(6): p. 791-795.

7. Esposito, M., C. Barausse, R. Pistilli, et al., *Short implants versus bone augmentation for placing longer implants in atrophic maxillae: One-year post-loading results of a pilot randomised controlled trial.* Eur J Oral Implantol, 2015. **8**(3): p. 257-68.

8. Felice, P., G. Cannizzaro, C. Barausse, et al., *Short implants versus longer implants in vertically augmented posterior mandibles: a randomised controlled trial with 5-year after loading follow-up.* Eur J Oral Implantol, 2014. **7**(4): p. 359-69.

9. Cannizzaro, G., P. Felice, M. Leone, et al., *Immediate versus early loading of 6.5 mm-long flapless-placed single implants: a 4-year after loading report of a split-mouth randomised controlled trial.* Eur J Oral Implantol, 2012. **5**(2): p. 111-21.

10. Esposito, M., G. Cannizarro, E. Soardi, et al., *A 3-year post-loading report of a randomised controlled trial on the rehabilitation of posterior atrophic mandibles: short implants or longer implants in vertically augmented bone?* Eur J Oral Implantol, 2011. **4**(4): p. 301-11.

11. Felice, P., G. Pellegrino, L. Checchi, et al., *Vertical augmentation with interpositional blocks of anorganic bovine bone vs. 7-mm-long implants in posterior mandibles: 1-year results of a randomized clinical trial.* Clin Oral Implants Res, 2010. **21**(12): p. 1394-403.

12. Cannizzaro, G., P. Felice, M. Leone, et al., *Early loading of implants in the atrophic posterior maxilla: lateral sinus lift with autogenous bone and Bio-Oss versus crestal mini sinus lift and 8-mm hydroxyapatite-coated implants. A randomised controlled clinical trial.* Eur J Oral Implantol, 2009. **2**(1): p. 25-38.

13. Cannizzaro, G., M. Leone, C. Torchio, et al., *Immediate versus early loading of 7-mm-long flapless-placed single implants: a split-mouth randomised controlled clinical trial.* Eur J Oral Implantol, 2008. **1**(4): p. 277-92.

14. Felice, P., G. Cannizzaro, V. Checchi, et al., *Vertical bone augmentation versus 7-mm-long implants in posterior atrophic mandibles. Results of a randomised controlled clinical trial of up to 4 months after loading.* Eur J Oral Implantol, 2009. **2**(1): p. 7-20.

15. Zygogiannis, K., I.H. Aartman, A. Parsa, et al., *Implant Mandibular Overdentures Retained by Immediately Loaded Implants: A 1-Year Randomized Trial Comparing the Clinical and Radiographic Outcomes Between Mini Dental Implants and Standard-Sized Implants.* Int J Oral Maxillofac Implants, 2017. **32**(6): p. 1377-1388.

16. Esposito, M., C. Barausse, R. Pistilli, et al., *Posterior jaws rehabilitated with partial prostheses supported by 4.0 x 4.0 mm or by longer implants: Four-month post-loading data from a randomised controlled trial.* Eur J Oral Implantol, 2015. **8**(3): p. 221-30.

17. Esposito, M., G. Cannizzaro, E. Soardi, et al., *Posterior atrophic jaws rehabilitated with prostheses supported by 6 mm-long, 4 mm-wide implants or by longer implants in augmented bone. Preliminary results from a pilot randomised controlled trial.* Eur J Oral Implantol, 2012. **5**(1): p. 19-33.

18. Felice, P., E. Soardi, G. Pellegrino, et al., *Treatment of the atrophic edentulous maxilla: short implants versus bone augmentation for placing longer implants. Five-month post-loading results of a pilot randomised controlled trial.* Eur J Oral Implantol, 2011. **4**(3): p. 191-202.

19. Zhang, X.M., J.Y. Shi, Y.X. Gu, et al., *Clinical Investigation and Patient Satisfaction of Short Implants Versus Longer Implants with Osteotome Sinus Floor Elevation in Atrophic Posterior Maxillae: A Pilot Randomized Trial.* Clin Implant Dent Relat Res, 2017. **19**(1): p. 161-166.

20. Esposito, M., G. Zucchelli, C. Barausse, et al., *Four mm-long versus longer implants in augmented bone in atrophic posterior jaws: 4-month post-loading results from a multicentre randomised controlled trial.* Eur J Oral Implantol, 2016. **9**(4): p. 393-409.

21. Felice, P., R. Pistilli, M. Piattelli, et al., *Posterior atrophic jaws rehabilitated with prostheses supported by 5 x 5 mm implants with a novel nanostructured calcium-incorporated titanium surface or by longer implants in augmented bone. Preliminary results from a randomised controlled trial.* Eur J Oral Implantol, 2012. **5**(2): p. 149-61.

22. Hsu, A., W.J. Seong, R. Wolff, et al., *Comparison of Initial Implant Stability of Implants Placed Using Bicortical Fixation, Indirect Sinus Elevation, and Unicortical Fixation.* Int J Oral Maxillofac Implants, 2016. **31**(2): p. 459-68.

23. Al-Hashedi, A.A., T.B. Taiyeb-Ali, and N. Yunus, *Outcomes of placing short implants in the posterior mandible: a preliminary randomized controlled trial.* Aust Dent J, 2016. **61**(2): p. 208-18.

24. Telleman, G., G.M. Raghoebar, A. Vissink, et al., *Impact of platform switching on peri-implant bone remodeling around short implants in the posterior region, 1-year results from a split-mouth clinical trial.* Clin Implant Dent Relat Res, 2014. **16**(1): p. 70-80.

25. Telleman, G., H.J. Meijer, A. Vissink, et al., *Short implants with a nanometer-sized CaP surface provided with either a platform-switched or platform-matched abutment connection in the posterior region: a randomized clinical trial.* Clin Oral Implants Res, 2013. **24**(12): p. 1316-24.

26. Grunder, U., G. Polizzi, R. Goené, et al., *A 3-year prospective multicenter follow-up report on the immediate and delayed-immediate placement of implants.* Int J Oral Maxillofac Implants, 1999. **14**(2): p. 210-6.

27. Telleman, G., G.M. Raghoebar, A. Vissink, et al., *Impact of platform switching on inter-proximal bone levels around 8.5 mm implants in the posterior region; 5-year results from a randomized clinical trial.* J Clin Periodontol, 2017. **44**(3): p. 326-336.

28. Öztürk, V., G. Emingil, N. Bostanci, et al., *Impact of implant-abutment connection on osteoimmunological and microbiological parameters in short implants: a randomized controlled clinical trial.* Clin Oral Implants Res, 2017. **28**(9): p. e111-e120.

29. Müller, F., E. Duvernay, A. Loup, et al., *Implant-supported mandibular overdentures in very old adults: a randomized controlled trial.* J Dent Res, 2013. **92**(12 Suppl): p. 154s-60s.

30. Schwindling, F.S., M. Raedel, N. Passia, et al., *The single mandibular implant study - Short-term effects of the loading protocol on Oral Health-related Quality of Life.* J Prosthodont Res, 2018. **62**(3): p. 313-316.

31. Schwarze, M., S. Budde, G. von Lewinski, et al., *No effect of conventional vs. minimally invasive surgical approach on clinical outcome and migration of a short stem total hip prosthesis at 2-year follow-up: A randomized controlled study.* Clin Biomech (Bristol, Avon), 2018. **51**: p. 105-112.

32. Blanco, J., A. Pico, L. Caneiro, et al., *Effect of abutment height on interproximal implant bone level in the early healing: A randomized clinical trial.* Clin Oral Implants Res, 2018. **29**(1): p. 108-117.

33. Nedir, R., N. Nurdin, S. Abi Najm, et al., *Short implants placed with or without grafting into atrophic sinuses: the 5-year results of a prospective randomized controlled study.* Clin Oral Implants Res, 2017. **28**(7): p. 877-886.

34. Rossi, F., D. Botticelli, G. Cesaretti, et al., *Use of short implants (6 mm) in a single-tooth replacement: a 5-year follow-up prospective randomized controlled multicenter clinical study.* Clin Oral Implants Res, 2016. **27**(4): p. 458-64.

35. Nedir, R., N. Nurdin, P. Khoury, et al., *Short Implants Placed with or without Grafting in Atrophic Sinuses: The 3-Year Results of a Prospective Randomized Controlled Study.* Clin Implant Dent Relat Res, 2016. **18**(1): p. 10-8.

36. Bergendal, T. and B. Engquist, *Implant-supported overdentures: a longitudinal prospective study.* Int J Oral Maxillofac Implants, 1998. **13**(2): p. 253-62.

37. Gunne, J., P. Astrand, T. Lindh, et al., *Tooth-implant and implant supported fixed partial dentures: a 10-year report.* Int J Prosthodont, 1999. **12**(3): p. 216-21.

38. de Molon, R.S., F.S. Lages, C.P. Rivera, et al., *Evaluation of Short and Regular Implants after Prosthesis Placement in the Mandible: A Nonrandomized Controlled Clinical Trial.* J Contemp Dent Pract, 2017. **18**(12): p. 1122-1129.
